# Association between severe drought and HIV prevention and care behaviors in Lesotho: A population-based survey 2016–2017

**DOI:** 10.1371/journal.pmed.1002727

**Published:** 2019-01-14

**Authors:** Andrea J. Low, Koen Frederix, Stephen McCracken, Salome Manyau, Elizabeth Gummerson, Elizabeth Radin, Stefania Davia, Herbert Longwe, Nahima Ahmed, Bharat Parekh, Sally Findley, Amee Schwitters

**Affiliations:** 1 ICAP at Columbia, Mailman School of Public Health, Columbia University, New York, New York, United States of America; 2 ICAP at Columbia Lesotho Office, Mailman School of Public Health, Columbia University, Maseru, Lesotho; 3 Division of Global HIV and TB, Centers for Disease Control and Prevention, Atlanta, Georgia, United States of America; 4 Centers for Disease Control and Prevention, Maseru, Lesotho; 5 ICAP at Columbia–Regional Office, Pretoria, South Africa; Africa Program, UNITED STATES

## Abstract

**Background:**

A previous analysis of the impact of drought in Africa on HIV demonstrated an 11% greater prevalence in HIV-endemic rural areas attributable to local rainfall shocks. The Lesotho Population-Based HIV Impact Assessment (LePHIA) was conducted after the severe drought of 2014–2016, allowing for reevaluation of this relationship in a setting of expanded antiretroviral coverage.

**Methods and findings:**

LePHIA selected a nationally representative sample between November 2016 and May 2017. All adults aged 15–59 years in randomly selected households were invited to complete an interview and HIV testing, with one woman per household eligible to answer questions on their experience of sexual violence. Deviations in rainfall for May 2014–June 2016 were estimated using precipitation data from Climate Hazards Group InfraRed Precipitation with Station Data (CHIRPS), with drought defined as <15% of the average rainfall from 1981 to 2016. The association between drought and risk behaviors as well as HIV-related outcomes was assessed using logistic regression, incorporating complex survey weights. Analyses were stratified by age, sex, and geography (urban versus rural). All of Lesotho suffered from reduced rainfall, with regions receiving 1%–36% of their historical rainfall. Of the 12,887 interviewed participants, 93.5% (12,052) lived in areas that experienced drought, with the majority in rural areas (7,281 versus 4,771 in urban areas). Of the 835 adults living in areas without drought, 520 were in rural areas and 315 in urban. Among females 15–19 years old, living in a rural drought area was associated with early sexual debut (odds ratio [OR] 3.11, 95% confidence interval [CI] 1.43–6.74, *p* = 0.004), and higher HIV prevalence (OR 2.77, 95% CI 1.19–6.47, *p* = 0.02). It was also associated with lower educational attainment in rural females ages 15–24 years (OR 0.44, 95% CI 0.25–0.78, *p* = 0.005). Multivariable analysis adjusting for household wealth and sexual behavior showed that experiencing drought increased the odds of HIV infection among females 15–24 years old (adjusted OR [aOR] 1.80, 95% CI 0.96–3.39, *p* = 0.07), although this was not statistically significant. Migration was associated with 2-fold higher odds of HIV infection in young people (aOR 2.06, 95% CI 1.25–3.40, *p* = 0.006). The study was limited by the extensiveness of the drought and the small number of participants in the comparison group.

**Conclusions:**

Drought in Lesotho was associated with higher HIV prevalence in girls 15–19 years old in rural areas and with lower educational attainment and riskier sexual behavior in rural females 15–24 years old. Policy-makers may consider adopting potential mechanisms to mitigate the impact of income shock from natural disasters on populations vulnerable to HIV transmission.

## Abstract 2

Andrea Low and colleagues present findings from their study in which a link between drought and increased HIV incidence in young girls in Lesotho is observed.

## Author summary

**Why was this study done?:**

- Periods of climate extremes have been shown to lead to increases in high-risk behaviors, particularly in agricultural communities dependent on rainfall for their livelihoods.
- Prior studies have linked these increases in riskier sexual behaviors, such as extramarital partnerships and transactional sex, to increases in HIV acquisition.
- The Lesotho Population-Based HIV Impact Assessment, a national HIV survey conducted from November 2016 to May 2017 following a 2-year severe drought in southern Africa, allowed us to reevaluate this relationship in the setting of expanded antiretroviral use.

**What did the researchers do and find?:**

- We paired geospatial data on accumulated rainfall from 2014 to 2016 with data from the survey to determine if there were any associations between drought and HIV outcomes.
- A total of 12,887 adults ages 15–59 years completed a detailed questionnaire, and 11,682 underwent an HIV test.
- Adolescent girls and young women ages 15–24 years in rural areas of drought had higher rates of high-risk behaviors, such as early sexual debut and transactional sex, and had lower educational attainment.
- Living in a drought area appeared to be associated with greater HIV prevalence in young females and was associated with a lower HIV prevalence in young males.
- However, external migration, commonly seen during these periods, was associated with a greater prevalence of HIV in men and women.

**What do these findings mean?:**

- Future policy on mitigation of climate change in southern Africa may consider including HIV prevention interventions in populations at high risk. This could include preexposure prophylaxis (PrEP) for migrants and young women in areas of severe food insecurity.
- Likewise, HIV programs that provide social and economic support to young women as part of an HIV prevention strategy should consider targeting areas affected by drought.
- Further studies should be done in other settings to investigate the external validity of these findings, particularly in terms of the impact on HIV in young women.

## Introduction

The impact of climate change on human health is becoming increasingly evident. Aside from changes in infectious disease transmission directly related to disturbances favoring multiplication of disease vectors, periods of climate extremes are often associated with changes in behavior as people struggle to survive in the face of loss of agricultural production [1,2]. As people, particularly women, address their food insecurity, they may be less likely to take steps to protect themselves from HIV infection [3,4], and studies have documented increases in HIV infections during drought-related famine periods in Africa [5,6]. A previous study of 21 Demographic and Health Surveys (DHS) across 19 countries in sub-Saharan Africa from 2003 to 2009 demonstrated that recurrent rainfall shocks were likely responsible for approximately 11% of HIV infections because of negative income shocks, particularly in high-prevalence countries and predominantly agrarian societies [6]. There is also concern that food insecurity could lead to decreased access to antiretrovirals (ARVs) because of economic constraints or decreased adherence or absorption of ARVs [7–9], with a subsequent increase in community viral load, drug resistance, and HIV transmission [10–13]. Despite the increased frequency and severity of droughts in the Sahel and southern Africa, few countries’ climate change adaptation policies currently include any intensified efforts for HIV prevention during climate-related events [14].

Southern Africa experienced 2 years of an El Nino–induced regional drought during the growing seasons of 2014–2015, including during key stages of crop development, leading to food shortages in 2016 and increased food costs for almost 40 million people in the region [15]. In Lesotho, where 55% of the population grow their own food, most people survive on rain-fed subsistence farming [16]. The drought led to a 67% reduction in maize production, with almost 25% of the population requiring emergency food assistance by August 2016 [16]. Lesotho also has a long tradition of labor migration to South Africa, and during periods of drought, migration increases, particularly from predominantly rural districts [16–18]. Furthermore, Lesotho is a country with a hyperendemic HIV epidemic, with prevalence above 25% in the adult population, and therefore is at greater risk of disruption to any improvements in epidemic control [3,19].

The Lesotho Population-Based HIV Impact Assessment (LePHIA) was a national survey conducted from November 2016 to May 2017 in collaboration with the Ministry of Health and the Centers for Disease Control and Prevention, with funding from the President’s Emergency Plan for AIDS Relief (PEPFAR). LePHIA examined the status of the HIV epidemic in Lesotho by measuring HIV prevalence, incidence, and viral load suppression (VLS). This study used LePHIA data to assess whether people living in areas most severely affected by the drought had higher HIV prevalence or changes in risk behaviors and whether there was any difference in the continuum of care among people living with HIV (PLHIV). The analysis additionally disaggregates by age band to examine the outcomes in youth and older people [19,20].

## Methods

### Survey design and participants

LePHIA employed a two-stage sampling design to select a nationally representative sample of adults and children aged 0–59 years in 418 enumeration areas (EAs) across all 10 districts. The sample size was powered on a relative standard error of 30% for incidence of HIV in adolescent girls and young women (AGYW). Consenting heads of household completed a household questionnaire, including a roster of all household members who resided in or had slept in the household the previous night. These individuals then consented to a questionnaire on sociodemographic and behavioral factors (S1 Text) and to home-based HIV testing. A guardian or parent provided permission to approach 10-to-17-year-olds who were then asked for assent for all procedures. The adult questionnaire was administered to participants aged 15–59. Written informed consent was documented at each stage via electronic signature. All participants provided written consent. The LePHIA protocol and data collection tools were approved by the Lesotho Research and Ethics Committee, the institutional review boards at Columbia University Medical Center (#AAAQ8537), and the United States Centers for Disease Control and Prevention.

### Procedures

Survey staff administered the household and the adult questionnaires during a face-to-face interview with participants using Google Nexus 9 tablets. The household questionnaire collected data on household assets and access to food. The adult questionnaire included questions on lifetime and recent sexual behaviors, as well as questions on the HIV continuum of care for those who reported being HIV positive. Only one female participant aged 15 years or older in each household was randomly selected to answer questions about experiences with sexual violence, to mask the nature of the questions to other members of the household. Any female younger than 18 who reported being sexually exploited was referred to support services for counseling and further management.

Rapid HIV testing was conducted using point-of-care (POC) tests—Determine HIV-1/2 Rapid Test (Alere)—and confirmed with a Uni-Gold HIV Test (Trinity Biotech), as per the national algorithm. Laboratory verification of all HIV-positive results was done using the Geenius HIV-1/2 Supplemental Assay (Bio-Rad). Viral load testing was done preferentially on plasma, or on dried blood spots (DBSs) if necessary, at a central lab on an automated platform.

### Drought measurement

Drought was quantified using precipitation estimates from the Climate Hazards Group InfraRed Precipitation with Station Data (CHIRPS), which blends a variety of satellite imagery with interpolated weather station data to create gridded rainfall estimates at dekadal time-step and 0.05° resolution, or approximately 30 km^2^ [21]. To capture the multiseason impact of El Nino, the 2-year total rainfall from June 2014 to May 2016 was summed and then ranked among all 2-year rainfall amounts within the 1981–2016 period and converted to an empirical percentile; therefore, a value of 1% signifies the driest 2-year period in the 35-year record, 100% signifies the wettest, and 50% signifies close to the median value. By using the rainfall accumulation over a 24-month period, the accumulation of data decreased the relative importance of errors in rainfall measurement. Use of these biennial deviations also ensured that any observed negative income shock for that period was a reflection of deviations from the norm, rather than a continuation of underlying farmers’ long-term adjustments to declining precipitation levels [21]. This gridded dataset was prepared by the Vulnerability Analysis and Mapping (VAM) Geospatial Analysis Team at the Analysis and Trends Service of the World Food Programme (WFP), using scripts developed in-house.

Latitude and longitude data from the centroid of each LePHIA-sampled EA were overlaid on the gridded rainfall dataset, with all individuals within each gridded area assigned the same level of rainfall, using ArcGIS Pro 2.0.1 (ESRI). Drought was defined at a percentile of 15% or lower of the historical record in order to generate a binary variable that approximates the level below which rainfall deficits are particularly harmful to gross domestic product (GDP) and maize yields [6]. This translates into the 2-year period being one of the five driest periods during the 35-year historical record.

### Statistical analysis

The effect of the drought was examined on recent behavior (over the past 12 months), HIV prevalence, and the continuum of HIV care at the individual level, using weighted data for all analyses, per an a priori analysis plan (S1 Analysis Plan). Commercial and forced sex were lifetime measures because of the smaller number of respondents. Design weights were calculated based on sampling design, including probability of household selection, and adjusted for nonresponse at the household, individual, and biomarker level using the Chi-Squared Automatic Interactor Detector (SI-CHAID) software (Statistical Innovations); this was stratified by urban or rural residence, age group, and region, with peri-urban populations grouped with rural as per the Lesotho Bureau of Statistics. Poststratification weights were calculated to reflect the age distribution of the 2016 Lesotho census. Additional weights were used for the subsample of women replying to the sexual violence questions. A household wealth quintile was generated using principal components analysis of household assets to generate a wealth score, based on the previously described methodology used in the DHS [22]. Poverty was defined as a household living in the lowest two quintiles. All analyses were done in Stata version 15.1 using weighted data, with Jackknife replicate weights used for variance estimation.

We used logistic regression to assess the association of drought with individual-level likelihood of infection as a binary variable, stratified by region and gender, based on prior data indicating that income shock differentially impacts males versus females [20,23], as does urban versus rural location [6]. The analysis was stratified by age to highlight the 15-to-24-year-olds and, where numbers allowed, into adolescents (aged 15–19 years) versus young adults (aged 20–24 years) to identify those most likely to have been recently infected. We examined the association with behaviors associated with HIV acquisition, such as recent condom use, commercial sex [24], and migration [25]; in young people for whom the drought would be most likely to affect recent transitions, we also examined associations with educational attainment [24], transactional and intergenerational sex (sexual partner who was 10 or more years older), and early sex and marriage in 15-to-19-year-olds [26,27]. Transactional sex was defined as nonmarital, noncommercial sex entered into on the assumption of material benefits [28]. Educational attainment was defined as whether they had attended secondary school or greater and did not require completion. Food insecurity was defined as any 24-hour period without food to eat because of lack of resources in the past 4 weeks. To assess drought and the treatment cascade, we assessed the association of drought with awareness of HIV status, reported ARV therapy (ART) use, and VLS, defined as HIV RNA < 1,000 copies/ml in PLHIV. Note that the planned investigation of the association of drought with recent infection was not performed because of the small number of recent cases identified in LePHIA.

A multivariable logistic regression model of the association of drought with HIV prevalence was constructed according to a UNAIDS hierarchical framework linking environmental disasters to population displacement, poverty, and behavioral change [3] and included variables known to be associated with HIV infection—namely, age and household economic status—or to be independently associated with drought in univariable analysis with a *p*-value < 0.10 and plausibly associated with HIV infection. Because there appeared to be opposite patterns of association between drought and HIV prevalence by sex, we fitted the model with an interactive variable combining drought and sex, with participants living without drought coded as 0, males living in drought coded as 1, and females living in drought coded as 2.

Because of the multiple comparisons included in this study, we used the Benjamini-Hochberg method to adjust the probabilities for the chance of a false positive [29]. For this calculation, we obtained the false discovery rate (FDR)-corrected *p*-value for each hypothesis and for each strata of analysis—for instance, including all univariable analyses conducted to assess the associations between drought and behavior in rural females. We used an acceptable level of false positives of 5% and report significance based on the corrected *p*-value, with a corrected *p*-value of 0.10 considered weak evidence of an association. For the multivariable analysis, we used a significance threshold of 0.025 based on the Bonferroni correction of adjusting the normal *p*-value of 0.05 to reflect the two models.

## Results

**Fig 1:**
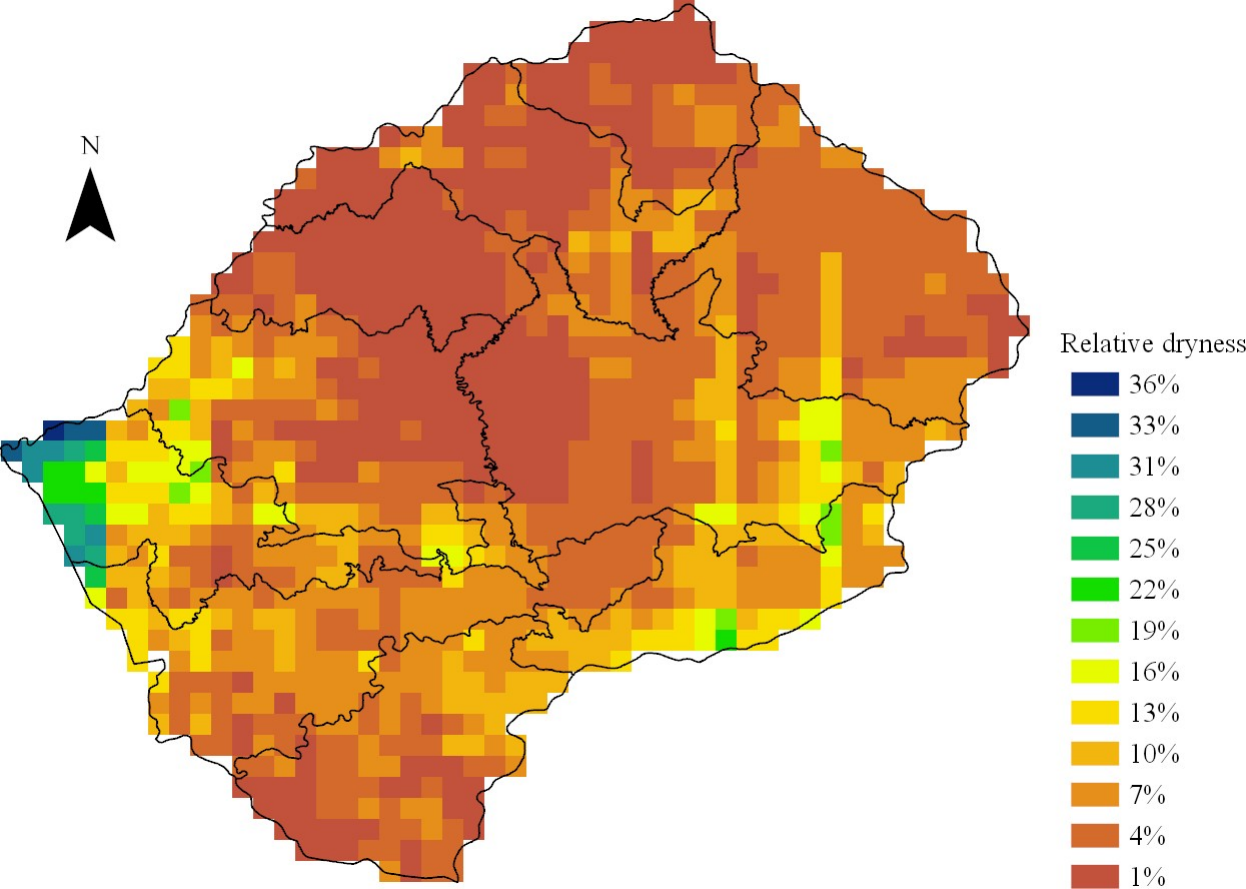
Map of precipitation in Lesotho during the drought of 2014–2016 relative to 1981–2016. Drought was defined as an empirical percentile of 15% or less of the historical record for the sum of rainfall from 2014 to 2016 compared to the sum for each 2-year period from 1981 to 2016. This translates into the 2-year period being one of the five driest periods during the 35-year historical record. *Source of precipitation grid: WFP, derived from CHIRPS satellite data [15].* CHIRPS, Climate Hazards Group InfraRed Precipitation with Station Data; WFP, World Food Programme.

Of 9,403 selected households, 8,824 (94%) completed a household interview; 9% of households in rural areas were vacant, compared to 6% of urban households. Of 7,893 eligible women and 6,135 eligible men, 12,887 (92%) completed an interview, and 11,682 of those (91%) were tested for HIV. The vast majority of households (94.8% of urban and 93.9% of rural) were in areas of drought (Fig 1). There was no difference in the sex ratio in drought versus nondrought areas, nor was there a difference in age distribution (Table 1). Compared to all urban residents and those living in rural nondrought areas, a greater proportion of participants in rural drought-affected areas were in impoverished (55.3%) and food-insecure households (39.6%).

**Table 1:** Selected characteristics of adult participants (15–59 years old) by residence and drought status, Lesotho 2016–2017.

	Urban		Rural		
Characteristic	No drought Weighted % (n) *N* = 315	Drought Weighted % (n) *N* = 4,771	No drought Weighted % (n) *N* = 520	Drought Weighted % (n) *N* = 7,281	Total Weighted % (n) *N* = 12,887
Sex					
Male	47.2% (125)	48.3% (1,894)	50.4% (219)	51.5% (3,123)	50.1% (5,361)
Female	52.8% (190)	51.7% (2,877)	49.7% (301)	48.5% (4,158)	49.9% (7,526)
Age (years)					
15–24	34.9% (115)	31.0% (1,520)	41.2% (214)	35.9% (2,571)	34.1% (4,420)
25–34	28.9% (82)	34.5% (1,594)	24.3% (118)	26.8% (1,854)	29.8% (3,648)
35–44	20.4% (64)	20.4% (934)	18.0% (94)	18.0% (1,302)	19.0% (2,394)
45–59	15.8% (54)	14.0% (723)	16.5% (94)	19.4% (1,554)	17.1% (2,425)
Household is impoverished^a^	16.1% (46)	9.4% (491)	25.2% (140)	55.3% (4,246)	35.2% (4,923)
Household is food insecure^b^	19.3% (58)	19.6% (949)	26.4% (146)	39.6% (2,964)	30.8% (4,117)
Lived outside Lesotho in past year	4.8% (17)	5.3% (244)	4.7% (24)	6.8% (477)	6.1% (762)
Currently or ever married	60.6% (190)	60.6% (2,898)	55.1% (299)	61.7% (4,680)	60.8% (8,067)
Secondary or higher education^c^	76.2% (243)	70.0% (3,330)	57.8% (294)	44.0% (3,131)	55.4% (6,998)

Percentages are survey weighted using Jackknife replicate weights. Note that totals might not add to 100%, because of rounding.

^a^ Defined as living in the lowest two wealth quintiles.

^b^ Defined as no food to eat in the household in the past 4 weeks because of lack of resources to get food.

^c^ Educational attainment is based on attendance and does not require completion.

In univariable analysis, both males and females residing in rural drought-affected areas were more likely to be poor, with the greatest effect on females, who had 4-fold higher odds of being members of impoverished households (odds ratio [OR] 4.22, 95% confidence interval [CI] 2.34–7.62, *p* < 0.001; Table 2). Among females residing in urban areas, drought was associated with an almost 5-fold increase in the odds of selling sex (OR 4.86, 95% CI 2.20–10.72, *p* < 0.001) and a 3-fold increase in the odds of having experienced forced sex (OR 3.11, 95% CI 1.42–6.85, *p* = 0.005). Among rural females, drought was associated with a reduction in condom use (OR 0.70, 95% CI 0.54–0.92, *p* = 0.01). Among males, there was weak evidence that living in rural drought-affected areas increased the odds of migration (OR 1.76, 95% CI 0.94–3.30, *p* = 0.08); this was not significant after FDR correction.

**Table 2:** Associations between drought and selected characteristics by residence and sex and on youths aged 15–24 years, Lesotho 2016–2017.

	Urban		Rural	
All adults (15–59 years)	Male OR (95% CI)^a^	Female OR (95% CI)^a^	Male OR (95% CI)^a^	Female OR (95% CI)^a^
Household is impoverished	0.53 (0.15–1.80) *p* = 0.31	0.56 (0.22–1.39) *p* = 0.21	3.24 (1.61–6.51) *p* = 0.001^f^	4.22 (2.34–7.62) *p* < 0.001^f^
Household is food insecure^b^	0.95 (0.48–1.90) *p* = 0.89	1.08 (0.53–2.22) *p* = 0.83	1.73 (0.91–3.30) *p* = 0.10	1.94 (1.10–3.43) *p* = 0.02^e^
Lived outside Lesotho in past year	1.47 (0.68–3.17) *p* = 0.32	0.85 (0.28–2.57) *p* = 0.77	1.76 (0.94–3.30) *p* = 0.08	1.20 (0.63–2.30) *p* = 0.57
Currently or ever married	1.01 (0.74–1.36) *p* = 0.97	0.99 (0.75–1.31) *p* = 0.96	1.40 (1.06–1.85) *p* = 0.02	1.23 (0.86–1.76) *p* = 0.25
Condom use at last sex	1.04 (0.75–1.45) *p* = 0.79	0.98 (0.64–1.48) *p* = 0.91	0.80 (0.60–1.08) *p* = 0.15	0.70 (0.54–0.92) *p* = 0.01^f^
Females only				
History of selling sex	NA	4.86 (2.20–10.72) *p* < 0.001^f^	NA	1.30 (0.70–2.44) *p* = 0.41
History of forced sex	NA	3.11 (1.42–6.85) *p* = 0.005^f^	NA	0.88 (0.58–1.34) *p* = 0.55
Youths (15–24 years)				
Migrated in past year	1.87 (0.74–4.71) *p* = 0.18	0.37 (0.11–1.27) *p* = 0.11	1.94 (0.56–6.78) *p* = 0.30	2.06 (0.29–14.66) *p* = 0.47
Secondary or higher education^c^	0.67 (0.31–1.45) *p* = 0.31	0.25 (0.06–1.14) *p* = 0.07	0.49 (0.22–1.05) *p* = 0.07	0.44 (0.25–0.78) *p* = 0.005^f^
Multiple partners in past year	2.23 (0.57–8.68) *p* = 0.25	1.62 (0.60–4.40) *p* = 0.34	1.63 (0.97–2.74) *p* = 0.06	0.50 (0.20–1.23) *p* = 0.13
Condom use at last sex	0.52 (0.11–2.37) *p* = 0.39	0.81 (0.25–2.64) *p* = 0.72	1.06 (0.55–2.04) *p* = 0.87	0.55 (0.31–0.99) *p* = 0.05
Females only				
Intergenerational sex in past year^d^	NA	2.34 (1.02–5.38) *p* = 0.05	NA	2.30 (0.84–6.35) *p* = 0.11
Transactional sex	NA	1.23 (0.52–2.94) *p* = 0.63	NA	3.26 (1.78–5.98) *p* < 0.001^f^
Age 15–19 only				
First sex before 15	0.39 (0.19–0.83) *p* = 0.01	4.28 (0.26–71.83) *p* = 0.31	1.73 (0.79–3.81) *p* = 0.17	3.11 (1.43–6.74) *p* = 0.004^f^
Currently or ever married	NA	0.74 (0.34–1.60) *p* = 0.45	0.88 (0.35–2.23) *p* = 0.79	7.33 (0.94–57.38) *p* = 0.06

Impoverished households are defined as living in the lowest two wealth quintiles.

^a^ OR calculated by logistic regression with Jackknife replicate variance estimates using weighted data.

^b^ Defined as no food to eat in the household in the past 4 weeks because of lack of resources to get food.

^c^ Defined as highest level attended.

^d^ Defined as sex with a partner older by 10 or more years.

^e^ A *p*-value that remained significant at the 0.10 level following FDR correction for multiple comparisons.

^f^ A *p*-value that remained significant at the 0.05 level following FDR correction for multiple comparisons.

Abbreviations: CI, confidence interval; FDR, false discovery rate; NA, numbers reporting are too small for analysis, or males were not asked; OR, odds ratio.

For young females living in a rural drought-affected area, there was an association with lower proportions attending secondary education (OR 0.44, 95% CI 0.25–0.78, *p* = 0.005), as well as a 3-fold increase in transactional sex (OR 3.26, 95% CI 1.78–5.98, *p <* 0.001). Among 15-to-19-year-olds, girls had increased odds of early sexual debut (OR 3.11, 95% CI 1.43–6.74, *p* = 0.004).

HIV prevalence was 26.9% (95% CI 25.4–33.4%) in urban areas and 24.7% (95% CI 23.5–25.8%) in rural areas and was higher in females (Table 3). Although the association was only significant at *p <* 0.10 after FDR correction, female adolescents living in drought-affected areas had almost 3-fold higher odds of HIV infection (OR 2.77, 95% CI 1.19–6.47, *p* = 0.02) compared to their counterparts in rural areas without drought. This association was weaker among girls living in urban drought areas (OR 1.84, 95% CI 0.94–3.62, *p* = 0.08), again compared to their counterparts in urban areas without drought.

**Table 3:** Associations between drought and HIV prevalence and care by residence, sex, and age, Lesotho 2016–2017.

	Urban		Rural	
	Male % (*n*/*N*)	Female % (*n*/*N*)	Male % (*n*/*N*)	Female % (*n*/*N*)
HIV prevalence	21.7% (379/1,709)	31.6% (903/2,729)	20.2% (640/3,053)	29.4% (1,277/4,191)
Prevalent HIV	OR (95% CI)^a^	OR (95% CI)^a^	OR (95% CI)^a^	OR (95% CI)^a^
Total	0.85 (0.52–1.40) *p* = 0.53	1.35 (0.87–2.08) *p* = 0.18	1.38 (0.77–2.46) *p* = 0.28	1.05 (0.79–1.38) *p* = 0.76
Age (years)				
15–24	0.48 (0.11–2.03) *p* = 0.32	1.62 (0.78–3.37) *p* = 0.20	approximately 1.00	0.88 (0.50–1.52) *p* = 0.63
15–19 (*N* = 2,077)	0.19 (0.02–1.71) *p* = 0.14	1.84 (0.94–3.62) *p* = 0.08	NA	2.77 (1.19–6.47) *p* = 0.02^b^
20–24 (*N* = 1,971)	0.73 (0.21–2.48) *p* = 0.61	1.36 (0.43–4.30) *p* = 0.60	NA	0.72 (0.44–1.18) *p* = 0.20
25–59 (*N* = 7,634)	0.91 (0.50–1.66) *p* = 0.75	1.19 (0.67–2.10) *p* = 0.54	1.07 (0.57–2.01) *p* = 0.83	1.07 (0.85–1.35) *p* = 0.56
HIV positive				
Aware of HIV status				
Total	0.92 (0.22–3.84) *p* = 0.90	0.55 (0.16–1.89) *p* = 0.34	1.19 (0.55–2.61) *p* = 0.66	1.30 (0.68–2.48) *p* = 0.43
Age (years)				
15–24 (*N* = 331)	NA	0.23 (0.08–0.70) *p* = 0.01^b^	NA	0.86 (0.14–5.15) *p* = 0.87
25–59 (*N* = 2,868)	0.59 (0.04–8.34) *p* = 0.69	0.62 (0.11–3.53) *p* = 0.59	0.92 (0.26–3.29) *p* = 0.90	1.30 (0.51–3.29) *p* = 0.58
VLS				
Of those on ART (*N* = 2,292)	2.00 (0.89–4.52) *p* = 0.09	1.91 (0.82–4.46) *p* = 0.13	1.42 (0.28–7.24) *p* = 0.67	0.90 (0.33–2.46) *p* = 0.83
Of all PLHIV	0.89 (0.43–1.87) *p* = 0.77	1.06 (0.60–1.88) *p* = 0.84	1.48 (0.69–3.14) *p* = 0.31	1.01 (0.62–1.65) *p* = 0.96
Age (years)				
15–24 (*N* = 330)	NA	0.32 (0.09–1.18) *p* = 0.09	NA	0.87 (0.17–4.60) *p* = 0.87
25–59 (*N* = 2,867)	0.28 (0.06–1.31) *p* = 0.11	0.88 (0.30–2.65) *p* = 0.83	0.61 (0.17–2.20) *p* = 0.45	1.48 (0.63–3.46) *p* = 0.37

Percentages are survey weighted using Jackknife replicate variance estimates.

^a^ OR calculated by logistic regression with Jackknife replicate variance estimates using weighted data.

^b^ A *p*-value that remained significant at the 0.10 level following FDR correction for multiple comparisons. There were no *p*-values significant at the 0.05 level following FDR correction.

Abbreviations: ART, antiretroviral therapy; CI, confidence interval; FDR, false discovery rate; NA, numbers too small for stratified analysis; OR, odds ratio; PLHIV, people living with HIV; VLS, viral load suppression.

In terms of the treatment cascade (Table 3), drought was not associated with awareness of status, reported ART use, or VLS among all PLHIV, aside from in young women in urban settings, for whom there was weak evidence of lower awareness of HIV-positive status (OR 0.23, 95% CI 0.08–0.70, *p* = 0.01).

In the multivariable analysis, after adjusting for age and economic, marital, and educational status as well as recent sexual behavior, there was a protective effect of drought noted for young males in terms of HIV infection (adjusted OR [aOR] 0.35, 95% CI 0.17–0.72, *p* = 0.006) and weak evidence of higher odds of HIV infection in young females (aOR 1.80, 95% CI 0.96–3.39, *p* = 0.07; Table 4). Recent migration, marital status, and intergenerational sex in the past year had the strongest associations with HIV in young people. In older people, there was no association between drought and HIV infection, but HIV infection was associated with food insecurity; being married; and reporting intergenerational, transactional, or commercial sex in the past year, whereas condom use at last sex was not protective. Higher educational attainment was protective in all ages.

**Table 4:** Associations between drought and related characteristics and HIV prevalence in 15-to-24- and 25-to-59-year-olds, Lesotho 2016–2017.

Characteristic	Ages 15–24 years *N* = 2,341 aOR (95% CI)^a^	Ages 25–59 years *N* = 5,778 aOR (95% CI)^a^
Gender–drought interaction^b^		
Males living in drought area	0.35 (0.17–0.72) *p* = 0.006^e^	0.72 (0.51–1.01) *p* = 0.06
Females living in drought area	1.80 (0.96–3.39) *p* = 0.07	1.18 (0.85–1.65) *p* = 0.31
Age (per 1-year increase)	1.16 (1.06–1.27) *p* = 0.002^e^	1.02 (1.01–1.02) *p* < 0.001^e^
Lives in rural community	0.64 (0.44–0.93) *p* = 0.02^e^	0.80 (0.68–0.94) *p* = 0.007^e^
Household is impoverished	0.68 (0.44–1.05) *p* = 0.08	0.96 (0.78–1.18) *p* = 0.70
Household is food insecure	1.24 (1.02–1.51) *p* = 0.03	1.22 (1.13–1.33) *p* < 0.001^e^
Lived outside Lesotho in past year	2.06 (1.25–3.40) *p* = 0.006^e^	1.12 (0.88–1.42) *p* = 0.34
Currently or ever married	1.74 (1.17–2.61) *p* = 0.009^e^	1.48 (1.18–1.86) *p* = 0.002^e^
Secondary or higher education^c^	0.66 (0.47–0.92) *p* = 0.02^e^	0.54 (0.46–0.62) *p* < 0.001^e^
Intergenerational sex in past year^d^	1.54 (0.97–2.44) *p* < 0.001^e^	1.37 (1.10–1.71) *p* = 0.007^e^
Transactional or commercial sex in past year	1.27 (0.63–2.56) *p* = 0.50	1.46 (1.10–1.93) *p* = 0.01^e^
Condom use at last sex	1.49 (0.99–2.24) *p* = 0.05	2.42 (2.13–2.76) *p* < 0.001^e^

All data are survey weighted.

^a^ aOR calculated by logistic regression with Jackknife replicate variance estimates using weighted data.

^b^ Reference group is anyone of either gender living in non-drought-affected areas.

^c^ Defined as highest level attended.

^d^ Defined as sex with a partner older by 10 or more years.

^e^ Significant using Bonferroni-corrected threshold for two models of 0.025.

Abbreviations: aOR, adjusted odds ratio; CI, confidence interval.

## Discussion

To our knowledge, this is the first study of the association of drought with HIV infection in the era of expanded use of ART [19]. In times of drought, it is expected that families will adopt extraordinary measures to ensure that they can secure food, potentially including such non-agricultural sources of income as casual labor, domestic service, and alternative forms of sex work. The LePHIA survey provided an opportunity to assess coping mechanisms that might have been adopted in the 12 months prior to the survey, during times of crop failure and hunger.

The findings from our study suggest a drought response pattern including alteration of behaviors increasing risk [6]. The observed associations were stronger in rural areas, where food shortages and income shocks would be most pronounced because of limited diversification of economic activity. They are consistent with other results from LePHIA, which found some association between food insecurity and incident infection in AGYW. Moreover, we found that there was an increase in the constellation of risk behaviors that were independently associated with HIV infection, including transactional and commercial sex, suggesting that some women may indeed be relying on sexual favors to survive drought, if less openly in rural areas than in urban [20,30]. The increase in early sexual debut and reduced educational attainment in girls in rural areas is consistent with adolescent girls being removed from school for transactional partnerships or marriage so that families can benefit from the bride price [31]. The strength of the association between marriage and HIV infection suggests that there may be relatively high rates of transmission between married couples, either through infections developed prior to marriage or through infections acquired from extramarital partners.

For women in urban areas, despite an attenuated effect of the drought on household poverty, being in a drought-affected region was associated with substantially higher reporting of commercial and coercive sex, which supports results from a recent United Nations Populations Fund survey in Lesotho linking drought to an increase in gender-based violence [32]. This is indicative of increased vulnerability, potentially reflective of internal migration from rural areas seeking employment, and of global disruption of the economy due to the severity of drought and impact on other sectors [33,34]. As 48% of women reporting a history of commercial sex work in Lesotho are HIV positive, and forced sex also increases the risk of HIV [19], the circular migration back to rural homesteads poses significant risk to partners and communities [35,36]. Furthermore, as education was strongly protective against HIV infection in our multivariable model, the lower school enrollment seen in young rural females in drought areas could have far-reaching consequences, in terms of both HIV acquisition and entrenchment of poverty. Drought clearly has both immediate and long-term consequences and requires different targeted policies.

Concerning the HIV continuum of care, the results are reassuring in terms of broader epidemic control, as there were no observed associations in terms of VLS. There were various interventions in the form of food support and household rations, including the Super Cereal from Global Fund and Ready-to-Use Therapeutic Food from the WFP, targeting malnourished PLHIV on ART or with tuberculosis, and these efforts may have been successful in incentivizing PLHIV to stay on treatment and in mitigating the worst hunger-associated effects of the drought. However, the highly migratory nature of the Lesotho population makes it difficult to interpret the completeness of the data; as those on ART were preferentially provided with food support [13], stable patients might have been more likely to remain in the country, with nonadherent or undiagnosed PLHIV not being captured by the survey because of out-migration.

Study limitations include the ecological nature of the study, which did not measure indicators of the experience of drought at the individual level. However, we were able to demonstrate that households in rural drought areas were significantly more likely to be impoverished, which we did not observe in urban areas, supporting the hypothesized impact of drought on agrarian households. Second, the timing of the survey, which was conducted after the drought, and its cross-sectional design limit the ability to determine causality for observed associations; however, the focus on behavior changes in the past year makes it more likely that observed behaviors occurred during the drought. Social desirability bias may have influenced reporting of sexual behaviors, although underreporting would be more likely to bias the results toward the null hypothesis. The severity of the drought also meant that few people were unaffected, limiting our ability to assess certain outcomes. Furthermore, the drought data were based on combined satellite and ground station measurement of rainfall, and there were only two ground stations in Lesotho, which had intermittent functionality during the drought period. This means that the drought grid was derived primarily from statistical downscaling of satellite data, impacting the accuracy of estimates at high resolution and allowing for some misclassification of drought severity. However, the use of 2 years of data and using relative rank rather than an absolute value for our indicator of drought should ensure that our results are reasonably robust. It should also be noted that the survey only included persons residing in Lesotho; those who had left and not returned as of the survey date may have influenced HIV transmission, but we could only observe the association between drought, migration, and HIV infection among returned migrants. Although there was only weak evidence of an association between migration and drought for rural males, the multivariable model identified a strong association between migration and HIV infection in young people. Migration has played a key role in the epidemic in the region, as male migrants have been reported to engage in commercial sex and have less access to HIV care [25,37,38]. The lower prevalence of HIV in young men suggests that, in the absence of migration, drought and its attendant financial constraints might protect men, as it may reduce both incentives and resources to pay for commercial or transactional sex [20]. Finally, because of the breadth of this analysis, the multiple comparisons increase the risk of type I errors, which we have tried to limit by using FDR. Some of the multivariable findings that were marginally significant before the correction become nonsignificant with the application of FDR. In part, this is due to the smaller number of cases once the data are disaggregated, limiting the statistical power to test multiple associations at once. Future work with larger samples of young people may enable more fine analyses of causality and the associations between risk factors. However, the positive and statistically significant associations between drought and risk behaviors among young women—and between those behaviors and HIV infection—remain, presenting a plausible and concerning pattern of vulnerability.

In conclusion, this study provides further evidence for the need for a coordinated policy and strategy to attenuate the effects of drought on HIV infection in southern Africa. Young women appear particularly susceptible to the negative income shocks of drought, whereas men seem predominantly affected in terms of labor migration and its potential for increased long-term risk [39]. Potential interventions should minimize these shocks by targeting the myriad factors contributing to vulnerability and could include cash transfers to encourage families to keep children in school and avoid early marriage, provided to rural families in times of food shortage [40,41], and expanded programs for AGYW, sex workers, and migrants, including preexposure prophylaxis (PrEP). In light of the anticipated acceleration of such climatic extremes, more research is urgently needed on improving resilience of crops to drought to mitigate the severity of impact on household incomes and public health.

## Supporting information

S1 Text LePHIA adult questionnaire. (PDF)

S1 Analysis Plan Project concept analysis plan. (DOCX)

S1 STROBE STROBE checklist. (DOC)

## Abbreviations

AGYW: adolescent girls and young women
aOR: adjusted odds ratio
ART: antiretroviral therapy
ARV: antiretroviral
CHIRPS: Climate Hazards Group InfraRed Precipitation with Station Data
CI: confidence interval
DBS: dried blood spot
DHS: Demographic and Health Survey
EA: enumeration area
FDR: false discovery rate
GDP: gross domestic product
LePHIA: Lesotho Population-Based HIV Impact Assessment
OR: odds ratio
PEPFAR: President’s Emergency Plan for AIDS Relief
PLHIV: people living with HIV
POC: point-of-care
PrEP: preexposure prophylaxis
SI-CHAID: Chi-Squared Automatic Interactor Detector
VAM: Vulnerability Analysis and Mapping
VLS: viral load suppression
WFP: World Food Programme
