# Supplementary material for: Association between severe drought and HIV prevention and care behaviors in Lesotho: A population-based survey 2016–2017
Source: PLoS Med. 2019 Jan 14;16(1):e1002727. doi: 10.1371/journal.pmed.1002727 (PMC6331084; doi:10.1371/journal.pmed.1002727)
Supplement: S1 Text — (PDF) [file pmed.1002727.s001.pdf]

LePHIA ADULT QUESTIONNAIRE

MODULE 1: RESPONDENT BACKGROUND

NO.	VARNAME	QUESTIONS	CODING CATEGORIES	SKIPS/FILTERS COMMENTS	INDICATOR	DHS/AIS	CORE
L1	LNGVQX_LNG	DO NOT READ:<br>LANGUAGE OF QUESTIONNAIRE	SESOTHO = 1<br>ENGLISH = 2		N/A	N/A	N/A
L2	LNGVINT_LNG	DO NOT READ:<br>LANGUAGE OF INTERVIEW	SESOTHO = 1<br>ENGLISH = 2<br><br>OTHER = 96<br>SPECIFY: ____________________		N/A	N/A	N/A
L3	LNGNAT_LNG	DO NOT READ:<br>NATIVE LANGUAGE OF PARTICIPANT	SESOTHO = 1<br>ENGLISH = 2<br><br>OTHER = 96<br>SPECIFY: ____________________		N/A	N/A	N/A
L4	TRNSLUSE	DO NOT READ:<br>TRANSLATION USED	YES = 1<br>NO = 2		N/A	N/A	N/A
		Thank you for agreeing to take part in this survey. The first set of questions is about your life in general. Afterwards, we will move on to other topics.			N/A	N/A	N/A

NO.	VARNAME	QUESTIONS	CODING CATEGORIES	SKIPS/FILTERS COMMENTS	INDICATOR	DHS/AIS	CORE
101	GENDR	DO NOT READ:<br>IS THE RESPONDENT MALE OR FEMALE?	MALE = 1<br>FEMALE = 2		N		
102	RELIGION	What is your religion?	ROMAN CATHOLIC = 1<br>LESOTHO EVANGELICAL = 2<br>ANGLICAN = 3<br>PENTECOSTAL = 4<br>OTHER CHRISTIAN = 5<br>OTHER RELIGION = 96<br>SPECIFY: _______________<br>DON'T KNOW = -8<br>REFUSED = -9				
103	SCHLAT	Have you ever attended school?	YES = 1<br>NO = 2<br>DON'T KNOW = -8<br>REFUSED = -9	NO, DK, REFUSED →WORK12MO	N	Y	C
104	SCHLHI	What is the highest level of school you attended: primary, secondary, or higher?	PRIMARY = 1<br>VOCATIONAL/TECHNICAL TRAINING AFTER PRIMARY = 2<br>SECONDARY/HIGH = 3<br>VOCATIONAL/TECHNICAL TRAINING AFTER SECONDARY/HIGH = 4<br>COLLEGE/UNIVERSITY = 5<br>GRADUATE/POST GRADUATE = 6<br>DON'T KNOW = -8<br>REFUSED = -9		N	Y	C

NO.	VARNAME	QUESTIONS	CODING CATEGORIES	SKIPS/FILTERS COMMENTS	INDICATOR	DHS/AIS	CORE
105	SCHCOM	What is the highest [standard/form/year] you <u>completed</u> at that level?<br><br>(IF ATTENDING FORM 1 OF SECONDARY SCHOOL AND HAS/DID NOT COMPLETE THE SCHOOL YEAR, ENTER “0”)	STANDARD/FORM/YEAR _____<br>DON’T KNOW = -8<br>REFUSED = -9		N	Y	C
FOLLOWING QUESTIONS ON SCHOOLING ARE FOR ADOLESCENT/YOUNG ADULTS AGES 15-18 – IF > 18, GO TO WORK12MO.							
106	SCHLCUR	Are you enrolled in school?	YES = 1<br>NO = 2<br>DON’T KNOW = -8<br>REFUSED = -9	DK, REFUSED →WORK12MO	N	N	C
107	ADCURGRD	What grade/form/year are you in now?	STANDARD/FORM/YEAR _____<br>DON’T KNOW = -8<br>REFUSED = -9		N	N	P

NO.	VARNAME	QUESTIONS	CODING CATEGORIES	SKIPS/FILTERS COMMENTS	INDICATOR	DHS/AIS	CORE
108	ADMISCH	During the last school week, did you miss any school days for any reason?	YES = 1<br>NO = 2<br>DON'T KNOW = -8<br>REFUSED = -9	NO, DK, REFUSED → ADCURGRD	N	N	P
109	ADMISCHREAS	Why did you miss school?	I HAVE BEEN SICK = 1<br>I DON'T FEEL SAFE TRAVELING TO SCHOOL = 2<br>I DON'T FEEL SAFE WHILE IN SCHOOL = 3<br>I DON'T LIKE SCHOOL = 4<br>I HAVE TO LOOK AFTER MY FAMILY = 5<br>THERE'S NOT ENOUGH MONEY TO SEND ME TO SCHOOL = 6<br>SCHOOL IS TOO FAR AWAY = 7<br>I HAVE TO WORK = 8<br>I HAVE A CHILD OR I AM PREGNANT (GIRLS ONLY) = 9<br>I MISSED TOO MUCH SCHOOL BECAUSE OF MY PERIOD (MENSTRUATION) (GIRLS ONLY) = 10<br>OTHER = 96<br>SPECIFY: ____________________<br>DON'T KNOW = -8<br>REFUSED = -9		N	N	P

NO.	VARNAME	QUESTIONS	CODING CATEGORIES	SKIPS/FILTERS COMMENTS	INDICATOR	DHS/AIS	CORE
110	ADLSTYRG RD	What grade/form/year were you in last year?	STANDARD/FORM/YEAR _______<br>DON'T KNOW = -8<br>REFUSED = -9	ALL → WORK12MO	N	N	P
111	ADNOSCH REAS	Why do you NOT go to school?	I HAVE BEEN SICK = 1<br>I DON'T FEEL SAFE TRAVELING TO SCHOOL = 2<br>I DON'T FEEL SAFE WHILE IN SCHOOL = 3<br>I DON'T LIKE SCHOOL = 4<br>I HAVE TO LOOK AFTER MY FAMILY = 5<br>THERE'S NOT ENOUGH MONEY TO SEND ME TO SCHOOL = 6<br>SCHOOL IS TOO FAR AWAY = 7<br>I HAVE TO WORK = 8<br>I HAVE A CHILD OR I AM PREGNANT (GIRLS ONLY) = 9<br>I MISSED TOO MUCH SCHOOL BECAUSE OF MY PERIOD (MENSTRUATION) (GIRLS ONLY) = 10<br>OTHER = 96<br>SPECIFY: ____________________<br>DON'T KNOW = -8<br>REFUSED = -9		N	N	P

NO.	VARNAME	QUESTIONS	CODING CATEGORIES	SKIPS/FILTERS COMMENTS	INDICATOR	DHS/AIS	CORE
112	ADLSTREG SCH	When was the last time you regularly attended school? Would you say it was less than a year ago or more than a year ago?	LESS THAN 1 YEAR = 1<br>1 YEAR OR LONGER = 2<br>DON'T KNOW = -8<br>REFUSED = -9		N	N	P
113	WORK12M O	Have you done any work in the last 12 months for which you received cash or goods as payment?	YES = 1<br>NO = 2<br>DON'T KNOW = -8<br>REFUSED = -9	NO, DK, REFUSED → SKIP TO END OF MODULE	N	Y (modified)	C
114	WORK7DA YS	Have you done any work in the last seven days for which you received cash or goods as payment?	YES = 1<br>NO = 2<br>DON'T KNOW = -8<br>REFUSED = -9		N	Y (modified)	C

NO.	VARNAME	QUESTIONS	CODING CATEGORIES	SKIPS/FILTERS COMMENTS	INDICATOR	DHS/AIS	CORE
115	WORKIND	What is your main product/service/activity of your place of work?	AGRICULTURE = 1<br>FISHING = 2<br>MINING/QUARRYING = 3<br>MANUFACTURING/PROCESSING = 4<br>ELECTRICITY = 5<br>CONSTRUCTION = 6<br>WHOLESALE & RETAIL, REPAIR OF MOTOR VEHICLES & PERSONAL & HOUSEHOLD GOODS = 7<br>HOTELS & RESTAURANTS = 8<br>TRANSPORT, STORAGE, & COMMUNICATION = 9<br>FINANCIAL INTERMEDIATION = 10<br>REAL ESTATE, RENDING & BUSSINESS ACTIVITIES = 11<br>PUBLIC ADMIN, DEFENCE, COMP. SOCIAL SERVICE = 12<br>EDUCATION = 13<br>HEALTH & SOCIAL WORK = 14<br>OTHER COMMUNITY, SOCIAL & PERS. SERVICES = 15<br>PRIVATE HOUSEHOLD = 16<br>EXTRA TERRITORIAL ORGANISATIONS BODIES = 17<br>OTHER = 96<br>SPECIFY = ______<br><br>DON'T KNOW = -8<br>REFUSED = -9				

MODULE 2: MOBILITY/MIGRATION

NO.	VARNAME	QUESTIONS	CODING CATEGORIES	SKIPS/FILTERS	INDICATOR	DHS/AIS	CORE
Now I would like to ask you questions about where you have lived.							

NO.	VARNAME	QUESTIONS	CODING CATEGORIES	SKIPS/FILTERS	INDICATOR	DHS/AIS	CORE
201	LIVETIME/LIVEUNITS	How long have you lived in Lesotho?<br><br>ONLY ONE OPTION MAY BE SELECTED. FOR EXAMPLE, ANSWER ONLY IN MONTHS OR IN YEARS.<br><br>CODE '0' IN MONTHS IF LESS THAN 1 MONTH.	MONTHS _____<br>YEARS _____<br>MY ENTIRE LIFE/BORN HERE= 96<br>DON'T KNOW = -8<br>REFUSE=-9	ENTIRE LIFE/BORN HERE/DK/REF → OUTSIDE	N	N	Country added
202	COUNTRY MVRSN	What was your main reason for moving to Lesotho?	TO JOIN FAMILY= 1<br>FOR MARRIAGE = 2<br>TO FIND WORK = 3<br>TO GO TO SCHOOL = 4<br>TO ESCAPE INSECURITY/CONFLICT = 5<br>TO ESCAPE DROUGHT, FLOOD, ETC= 6<br>TO ESCAPE VIOLENCE IN HOUSEHOLD = 7<br>OTHER = 96<br>SPECIFY: ____________________<br><br>DON'T KNOW =-8<br>REFUSED =-9		N	N	Country added
203	OUTSIDE	Have you ever lived outside Lesotho?<br><br>.	YES = 1<br>NO = 2<br><br>DON'T KNOW = -8<br>REFUSED = -9	IF NO, DK, REFUSED → END OF MODULE	N	N	Country added
204	OUTFIRST	How old were you the first time you lived outside Lesotho?	AGE IN YEARS ___ ___<br><br>DON'T KNOW = -8<br>REFUSED = -9		N	N	Country added

NO.	VARNAME	QUESTIONS	CODING CATEGORIES	SKIPS/FILTERS	INDICATOR	DHS/AIS	CORE
205	OUTNUMBER	How many times in your life have you ever lived outside of Lesotho?	NUMBER OF TIMES ______<br><br>DON'T KNOW = -8<br>REFURSED = -9		N	N	Country added
206	LONGPER	What was the longest period of time you spent living outside Lesotho since you were 18 years old?	WEEKS __ __<br>MONTHS __ __<br>YEARS __ __<br><br>DON'T KNOW = -8<br>REFUSED = -9		N	N	Country added
207	12MONTHOUT	In the last 12 months, have you been away from home for more than one month at a time?	YES = 1<br>NO = 2<br><br>DON'T KNOW = -8<br>REFUSED = -9	NO, DK, REFUSED → END OF MODULE			Country added
208	REASONAWAY	For what reason were you away from home for longer than one month?	WORK = 1<br>SCHOOL = 2<br>FAMILY OBLIGATIONS = 3<br>MEDICAL CARE = 4<br>TRAVEL = 5<br>OTHER = 6<br>SPECIFY = _____<br><br>DON'T KNOW = -8<br>REFUSED = -9				Country added
209	LOCAWAY	In what country/countries did you spend more than one month living in during the past year?	SOUTH AFRICA = A<br>SWAZILAND = B<br>MOZAMBIQUE = C<br>NAMIBIA = D<br>BOTSWANA = E<br>ZIMBABWE = F<br>OTHER = G<br>SPECIFY = ______<br><br>DON'T KNOW = Y<br>REFUSED = Z				Country added

NO.	VARNAME	QUESTIONS	CODING CATEGORIES	SKIPS/FILTERS	INDICATOR	DHS/AIS	CORE
210	WORKIND OUT	What was your main product/service/activity of your place of work the last time you lived outside of Lesotho?	DID NOT WORK = 0<br>AGRICULTURE = 1<br>FISHING = 2<br>MINING/QUARRYING = 3<br>MANUFACTURING/PROCESSING = 4<br>ELECTRICITY = 5<br>CONSTRUCTION = 6<br>WHOLESALE & RETAIL, REPAIR OF MOTOR VEHICLES & PERSONAL & HOUSEHOLD GOODS = 7<br>HOTELS & RESTAURANTS = 8<br>TRANSPORT, STORAGE, & COMMUNICATION = 9<br>FINANCIAL INTERMEDIATION = 10<br>REAL ESTATE, RENDING & BUSS. ACTIVITIES = 11<br>PUBLIC ADMIN, DEFENCE, COMP. SOCIAL SERVICE = 12<br>EDUCATION = 13<br>HEALTH & SOCIAL WORK = 14<br>OTHER COMMUNITY, SOCIAL & PERS. SERVICES = 15<br>PRIVATE HOUSEHOLD = 16<br>EXTRA TERRITORIAL ORGANISATIONS BODIES = 17<br>OTHER = 96<br>SPECIFY = ______<br><br>DON'T KNOW = -8<br>REFUSED = -9				Country added

MODULE 3: MARRIAGE

NO.	VARNAME	QUESTIONS	CODING CATEGORIES	SKIPS/FILTERS	INDICATOR	DHS/AIS	CORE
Now I would like to ask you about your current and previous relationships and/or marriages.							

NO.	VARNAME	QUESTIONS	CODING CATEGORIES	SKIPS/FILTERS	INDICATOR	DHS/AIS	CORE
301	EVERMAR	Have you ever been married or lived together with a [man/woman] as if married?	YES = 1<br>NO = 2<br>DON'T KNOW =-8<br>REFUSED = -9	NO, DK, REFUSED → SKIP TO END OF MODULE	N	Y	C
302	AGEMAR	How old were you the first time you married or started living with a [man/woman] as if married?	AGE IN YEARS ___ ___<br><br>DON'T KNOW = -8<br>REFUSED = -9		N	Y (modified)	S
303	CURMAR	What is your marital status now: are you married, living together with someone as if married, widowed, divorced, or separated?	MARRIED = 1<br>LIVING TOGETHER = 2<br>WIDOWED = 3<br>DIVORCED = 4<br>SEPARATED = 5<br>DON'T KNOW = -8<br>REFUSED = -9	WIDOWED, DIVORCED, SEPARATED, DK, REFUSED → SKIP TO END OF MODULE	N	N	C
The next several questions are about your current spouse or partner(s).							
304	NUMWIF	Altogether, how many wives or live-in partners do you have?	NUMBER OF WIVES OR LIVE-IN PARTNERS _____<br>DON'T KNOW = -8<br>REFUSED = -9	DK, REFUSED →SKIP TO END OF MODULE<br><br>SKIP IF FEMALE	N	Y	C
305	QXA1205_LIST/QXA1205	The Household Schedule listed [INSERT NUMBER OF REPORTED PARTNERS] household members as your wives/partners. Please review the list below. Are all of the listed household members your wives/partners who live in the household?	YES = 1<br>NO = 2	NO→ NPYN<br><br>SKIP IF FEMALE	N	N	C

NO.	VARNAME	QUESTIONS	CODING CATEGORIES	SKIPS/FILTERS	INDICATOR	DHS/AIS	CORE
306	REVWIFENM	Is [HHRNAME**] your wife/partner?	YES = 1<br>NO = 2	SKIP IF FEMALE	N	N	C
307	REVWIFEHH	Does [HHRNAME**] live in the household?	YES = 1<br>NO = 2	SKIP IF FEMALE	N	N	C
308	NPYN	Do you have additional spouse(s)/partner(s) that live with you?	YES = 1<br>NO = 2	SKIP IF FEMALE	N	N	C
309	NPNUM	How many additional spouse(s)/partners(s) live with you?	NUMBER OF SPOUSES OR LIVE-IN PARTNERS _ _	SKIP IF FEMALE	N	N	C
310	NEWHNAME	Please enter the name of your spouse/partner that lives with you.	NAME OF SPOUSE/PARTNER ____<br>DON'T KNOW = -8<br>REFUSED = -9	SKIP IF FEMALE	N	N	C
311	WIFLIVEEW	How many wives or live-in partners do you have who live elsewhere?	NUMBER OF ADDITIONAL SPOUSE(S)/PARTNERS ____<br>DON'T KNOW = -8<br>REFUSED = -9	SKIP IF FEMALE	N	Y	C
312	HUSLIVEW	Is your husband or partner living with you now or is he staying elsewhere?	LIVING TOGETHER = 1<br>STAYING ELSEWHERE = 2<br>DON'T KNOW = -8<br>REFUSE TO ANSWER = -9	STAYING ELSEWHERE, DK, REFUSED →HUSOTWIF	N	Y	C

NO.	VARNAME	QUESTIONS	CODING CATEGORIES	SKIPS/FILTERS	INDICATOR	DHS/AIS	CORE
				STAYING ELSEWHERE & LISTED PARTNER IN HH ROSTER→ HHQXHUS2<br><br>SKIP IF MALE			
313	HHQXHUS2	The household schedule listed [NAME OF HUSBAND/PARTNER] as your husband/partner who is living here. Is that correct?	YES = 1<br>NO = 2<br>DON'T KNOW = -8<br>REFUSED = -9	YES<br>DK, REF→HUSOTWIF<br><br>SKIP IF MALE	N	N	C
314	NEWHSELECT	Please select the spouse/partner that lives with you.	[LIST OF PERSONS ON HH ROSTER]<br>NOT LISTED IN HOUSEHOLD = 96	LISTED →HUSOTWIF<br><br>SKIP IF MALE	N	N	C
315	NEWHNAME	Please enter the name of your spouse/partner that lives with you.	NAME OF SPOUSE/PARTNER ____<br>DON'T KNOW = -8<br>REFUSED = -9	SKIP IF MALE	N	N	C
316	HUSOTWIF	Does your husband or partner have other wives or does he live with other women as if married?	YES = 1<br>NO = 2<br>DON'T KNOW = -8<br>REFUSE TO ANSWER = -9	NO, DK, REFUSED→ SKIP TO END OF MODULE<br><br>SKIP IF MALE	N	Y(modified)	C

NO.	VARNAME	QUESTIONS	CODING CATEGORIES	SKIPS/FILTERS	INDICATOR	DHS/AIS	CORE
317	HUSNWIF	Including yourself, in total, how many wives or live-in partners does your husband or partner have?	NUMBER OF WIVES OR LIVE-IN PARTNERS _ _<br><br>DON'T KNOW = -8<br>REFUSE TO ANSWER = -9	SKIP IF MALE	N	Y(modified)	C

MODULE 4: REPRODUCTION

NO.	VARNAME	QUESTIONS	CODING CATEGORIES	SKIPS/FILTERS	INDICATOR	DHS/AIS	CORE
Now I would like to ask you questions about your pregnancies and your children.				IF MALE SKIP TO AVOIDPREG.			
401	PREGNUM	How many times have you been pregnant including a current pregnancy?<br><br>CODE '00' IF NONE.	NUMBER OF TIME(S) ____<br>DON'T KNOW = -8<br>REFUSED = -9	NONE, DK, REFUSED → AVOIDPREG	N	N	C
402	LIVEB	Have you ever had a pregnancy that resulted in a live birth?<br><br>A live birth is when the baby shows signs of life, such as breathing, beating of the heart or movement.	YES = 1<br>NO = 2<br>DON'T KNOW = -8<br>REFUSED = -9	NO, DK, REFUSED →PREGNANT	N	N	C
403	TOTCHILD NUM	In total, how many children have you given birth to who were born alive?<br><br>These include children who were born alive but later died. They could have been children who have lived with you or have not lived with you.	NUMBER OF CHILDREN ____<br>DON'T KNOW = -8<br>REFUSED = -9	NONE, DK, REFUSED→ CHILDA2012	N	N	S

NO.	VARNAME	QUESTIONS	CODING CATEGORIES	SKIPS/FILTERS	INDICATOR	DHS/AIS	CORE
404	CHILDA2012	How many live births have you had since the **1st of January, 2013**?<br><br>CODE ‘00’ IF NONE.	NUMBER OF CHILDREN ____<br>DON’T KNOW = -8<br>REFUSED = -9	NONE, DK, REFUSED →PREGNANT<br><br>YEAR IS SURVEY YEAR – 3 YEARS	N	N	C

NO.	VARNAME	QUESTIONS	CODING CATEGORIES	SKIPS/FILTERS	INDICATOR	DHS/AIS	CORE
Now I would like to ask you some questions about the <u>last</u> pregnancy that resulted in a live birth since the 1st of January **2013**].							
405	PRGTWIN	Did your last pregnancy result in birth to twins or more?	YES = 1<br>NO = 2<br>DON’T KNOW = -8<br>REFUSED = -9	NO, DK, REF →CHILDLAST	N	N	C
	PRGTWINNAME*<br><br>PRGTWINPLUS	What is the name of the [BIRTHORDER*] born child from your last pregnancy that resulted in a live birth?<br>A live birth is when the baby shows signs of life, such as breathing, beating of the heart or movement.<br><br>DO NOT READ: IF THE CHILD WAS NOT NAMED BEFORE DEATH, INPUT BIRTH AND THE BIRTH ORDER NUMBER. FOR EXAMPLE, "BIRTH 1".	NAME ________	WILL BE REPEATED FOR EACH MULTIPLE BIRTH	N	N	C

NO.	VARNAME	QUESTIONS	CODING CATEGORIES	SKIPS/FILTERS	INDICATOR	DHS/AIS	CORE
		DO NOT READ: WAS THERE ANOTHER MULTIPLE BORN ALIVE?					
406	CHILDLAST	What is the name of the child from your last pregnancy that resulted in a live birth?<br><br>A live birth is when the baby shows signs of life, such as breathing, beating of the heart or movement.<br><br>IF THE CHILD WAS NOT NAMED BEFORE DEATH, INPUT BIRTH 1.	NAME ______<br>ID NUMBER______		N	N	C
407	PRGPLAN	When you were pregnant with [CHILDLAST/PRGTWINNAME*], did you plan to get pregnant at that time?	YES = 1<br>NO = 2<br>DON'T KNOW = -8<br>REFUSED = -9	YES, NO,DK, REFUSED → PRGCARE	N	Y	S
408	PRGCARE	When you were pregnant with **[CHILDLAST/PRGTWINNAME*]**, did you visit a health facility for antenatal care?	YES = 1<br>NO = 2<br>DON'T KNOW = -8<br>REFUSED = -9	NO, DK, REFUSED → PREGNCR	N	Y(AIS)	C
409	PRGMONTHS	At what months in your pregnancy [CHILDLAST/PRGTWINNAME*] did you first attend the antenatal clinic?<br><br>DO NOT READ: SELECT ALL THAT APPLY. SHOW AID IF UNSURE	0-3 MONTHS/1ST TRIMESTER = A<br>4-6 MONTHS/2ND TRIMESTER = B<br>7-9 MONTHS/3RD TRIMESTER = C<br>DON'T KNOW = Y<br>REFUSED = Z	ELECTRONIC AID IF DON'T KNOW	N	N	S
410	PREGNCR	What is the <u>main</u> reason you did not visit a clinic for antenatal care when you were pregnant with **[CHILDLAST/PRGTWINNAME*]**?	CLINIC WAS TOO FAR AWAY = 1<br>COULD NOT TAKE TIME OFF WORK/TOO BUSY = 2<br>COULD NOT AFFORD TO PAY FOR THE VISIT = 3	ALL→ BRTHWHR<br><br>ADAPT RESPONSES TO LOCAL	N	N	C

NO.	VARNAME	QUESTIONS	CODING CATEGORIES	SKIPS/FILTERS	INDICATOR	DHS/AIS	CORE
			DID NOT TRUST THE CLINIC STAFF = 4<br>RECEIVED CARE AT HOME = 5<br>DID NOT WANT AN HIV TEST DONE = 6<br>HUSBAND/FAMILY WOULD NOT LET ME GO = 7<br>USED TRADITIONAL BIRTH ATTENDANT/HEALER = 8<br>COST OF TRANSPORT = 9<br>RELIGIOUS REASONS = 10<br>OTHER = 96<br>SPECIFY: ____________________<br>DON'T KNOW = -8<br>REFUSED = -9	CONTEXT.			
QX1601D	I will now be asking you questions on HIV testing. Please remember that your responses will be kept confidential and will not be shared with anyone else.						
411	HIVTSBP	Have you ever tested for HIV before your pregnancy with **[CHILDLAST/PRGTWINNAME*]**?	YES = 1<br>NO = 2<br>DON'T KNOW = -8<br>REFUSED = -9	NO, DK, REFUSED → HIVTOPG	N	N	C
412	HIVPSBP	Did you test positive for HIV before your pregnancy with [CHILDLAST/PRGTWINNAME*]?	YES = 1<br>NO = 2<br>DON'T KNOW = -8<br>REFUSED = -9	NO, DK, REFUSED→ HIVTOPG	N	N	C
413	ARVFVST	At the time of your first antenatal care visit when you were pregnant with **[CHILDLAST/PRGTWINNAME*]**, were you	YES = 1<br>NO = 2<br>DON'T KNOW = -8	YES →BIRTHWHR<br>NO, DK,	N	N	C

NO.	VARNAME	QUESTIONS	CODING CATEGORIES	SKIPS/FILTERS	INDICATOR	DHS/AIS	CORE
		taking ARVs, that is, antiretroviral medications to treat HIV?	REFUSED = -9	REFUSED→ ARVTKPG<br><br>ELECTRONIC AID IF DON'T KNOW			
414	HIVTOPG	During any of your visits to the antenatal care clinic when you were pregnant with **[CHILDLAST/PRGTWINNAME*],** were you <u>offered</u> an HIV test?	YES = 1<br>NO = 2<br>DON'T KNOW = -8<br>REFUSED = -9		N	Y(AIS)	C
415	HIVTRPRG	Were you <u>tested</u> for HIV during any of your antenatal care clinic visits when you were pregnant with **[CHILDLAST/PRGTWINNAME*]**?	YES = 1<br>NO = 2<br>DON'T KNOW = -8<br>REFUSED = -9	YES →HIVRTPG<br>DK, REFUSED →BRTHWHR	N	N	C
416	HIVTSNR	What is the main reason you were not tested for HIV during antenatal care with **[CHILDLAST/PRGTWINNAME*]**?	DID NOT WANT AN HIV TEST DONE / DID NOT WANT TO KNOW MY STATUS = 1<br>DID NOT RECEIVE PERMISSION FROM SPOUSE/FAMILY = 2<br>AFRAID OTHERS WOULD KNOW ABOUT TEST RESULTS = 3<br>DID NOT NEED TEST/LOW RISK = 4<br>OTHER = 96<br>SPECIFY: ____________________<br>DON'T KNOW = -8<br>REFUSED = -9	ALL→SKIP TO BIRTHWHR<br><br>ADAPT RESPONSES BASED ON LOCAL CONTEXT.	N	N	C
417	HIVRTPG	What was the result of your last HIV test during your pregnancy with **[CHILDLAST/PRGTWINNAME*]**?	POSITIVE = 1<br>NEGATIVE = 2<br>UNKNOWN/INDETERMINATE = 3<br>DID NOT RECEIVE RESULTS = 4<br>DON'T KNOW = -8	NEGATIVE, UNK, NO RESULTS, DK, REF →	N	N	C

NO.	VARNAME	QUESTIONS	CODING CATEGORIES	SKIPS/FILTERS	INDICATOR	DHS/AIS	CORE
			REFUSED = -9	BIRTHWHR			
418	ARVTKPG	Did you take ARVs during your pregnancy with [NAME] to stop **[CHILDLAST/PRGTWINNAME*]** from getting HIV?	YES = 1<br>NO = 2<br>DON'T KNOW = -8<br>REFUSED = -9	YES, DK, REFUSED → BIRTHWHR	N	N	C
419	ARVNRPG	What was the main reason you did not take ARVs while you were pregnant with **[CHILDLAST/PRGTWINNAME*]**?	WAS NOT PRESCRIBED = 1<br>I FELT HEALTHY/NOT SICK = 2<br>COST OF MEDICATIONS = 3<br>COST OF TRANSPORT = 4<br>RELIGIOUS REASONS = 5<br>WAS TAKING TRADITIONAL MEDICATIONS = 6<br>MEDICATIONS OUT OF STOCK = 7<br>DID NOT WANT PEOPLE TO KNOW HIV STATUS = 8<br>DID NOT RECEIVE PERMISSION FROM SPOUSE/FAMILY = 9<br>OTHER = 96<br>SPECIFY: ____________________<br>DON'T KNOW = -8<br>REFUSED = -9		N	N	C
420	BIRTHWHR	Where did you give birth to **[CHILDLAST/PRGTWINNAME*]**?	AT HOME = 1<br>AT A HEALTH FACILITY = 2<br>IN TRANSIT = 3<br>OTHER = 96<br>SPECIFY: ____________________<br>DON'T KNOW = -8<br>REFUSED = -9	HOME, TRANSIT, OTH, DK, REFUSED → CHILDBDATE	N	Y(MODIFIED)	C

NO.	VARNAME	QUESTIONS	CODING CATEGORIES	SKIPS/FILTERS	INDICATOR	DHS/AIS	CORE
421	BIRTHFAC	What health facility or hospital did you deliver [CHILDLAST/PRGTWINNAME*] at?	PICK FROM LIST<br>ENTER NAME __________________<br>DON'T KNOW = -8<br>REFUSED = -9				
422	HIVTOBR	Were you offered an HIV test during labor?	YES = 1<br>NO = 2<br>DON'T KNOW = -8<br>REFUSED = -9		N	Y(MODIFIED)	C
423	HIVTTLB	Did you test for HIV during labor?	YES = 1<br>NO = 2<br>DON'T KNOW = -8<br>REFUSED = -9	NO, DK, REFUSED → CHILDBDATE<br><br>SKIP IF HIV POSITIVE	N	N	C
424	HIVRSLR	What was the result of that test?	POSITIVE = 1<br>NEGATIVE = 2<br>UNKNOWN/INDETERMINATE = 3<br>DID NOT RECEIVE RESULTS = 4<br>DON'T KNOW = -8<br>REFUSED = -9	NEG, UNK/INDET,NO RESULTS, DK, REFUSED → CHILDBDATE<br><br>SKIP IF HIV POSITIVE	N	N	C
425	ARVOFLB	During labor, were you offered ARVs to protect [NAME] against HIV?<br><br>DO NOT READ: SHOW ARV GRAPHIC IF PARTICIPANT IS UNSURE	YES = 1<br>NO = 2<br>DON'T KNOW = -8<br>REFUSED = -9	SKIP IF ALREADY ON ARVS.	N	N	C
426	ARVTKLB	During labor, did you take ARVs to protect **[CHILDLAST/PRGTWINNAME*]** against HIV?<br><br>DO NOT READ: SHOW ARV GRAPHIC IF PARTICIPANT IS UNSURE	YES = 1<br>NO=2<br>DON'T KNOW = -8<br>REFUSED = -9	NO, DK, REFUSED→ CHILDBDATE<br><br>ADAPT BASED ON COUNTRY CONTEXT.	N	N	C

NO.	VARNAME	QUESTIONS	CODING CATEGORIES	SKIPS/FILTERS	INDICATOR	DHS/AIS	CORE
				ELECTRONIC AID IF DON'T KNOW.<br><br>SKIP IF ALREADY ON ARVS.			
427	ARVCNTN	Did you continue to take ARVs after delivery?	YES = 1<br>NO= 2<br>DON'T KNOW =8<br>REFUSED = -9	ADAPT TO LOCAL COUNTRY CONTEXT.<br><br>SKIP IF ALREADY ON ARVS.	N	N	C
428	CONTARV	For how many months after delivery did you continue to take ARVs?	MONTH __ __<br>STILL TAKING ARVS = 95<br><br>DON'T KNOW = -8<br>REFUSED = -9		N	N	Country Added
429	CHILDBDA TE	When did you give birth to **[CHILDLAST/PRGTWINNAME*]**? Please give your best guess.	DAY __ __<br>DON'T KNOW DAY= -8<br>REFUSED DAY= -9<br><br>MONTH __ __<br>DON'T KNOW MONTH= -8<br>REFUSED MONTH= -9<br><br>YEAR __ __ __ __<br>DON'T KNOW YEAR=-8<br>REFUSED YEAR= -9		N	N	C

NO.	VARNAME	QUESTIONS	CODING CATEGORIES	SKIPS/FILTERS	INDICATOR	DHS/AIS	CORE
430	CHILDALIVE	Is **[CHILDLAST/PRGTWINNAME*]** still alive?	YES = 1<br>NO = 2<br>DON'T KNOW = -8<br>REFUSED = -9	YES, DK, REFUSED →CHILDLIVE<br>NO→CHILDDDATE	N	N	C
431	CHILDDDATE	How old was **[CHILDLAST/PRGTWINNAME*]** when he/she died?<br><br>KEY '0' IF CHILD WAS LESS THAN ONE YEAR OLD	COMPLETED YEARS _____<br>DON'T KNOW = -8<br>REFUSED = -9	>0, DK, REF →CHILDDATE	N	N	C
432	CHILDDDATE	How old was **[CHILDLAST/PRGTWINNAME*]** in months when he/she died?<br><br>KEY '0' IF LESS THAN ONE MONTH OLD.	COMPLETED MONTHS ______<br>DON'T KNOW = -8<br>REFUSED = -9		N	N	C
433	CHILDLIVE	Is **[CHILDLAST/PRGTWINNAME*]** living with you?	YES = 1<br>NO = 2	NO →CHILDBF	N	N	C
434	CHILDHHNUM	Please select [CHILDLAST/PRGTWINNAME*] that lives with you.<br><br>RECORD '0' IF CHILD NOT LISTED IN HOUSEHOLD	[LIST OF CHILDREN IN HOUSEHOLD]<br>NOT LISTED IN HOUSEHOLD = 96		N	N	C
435	CHILDBF	Did you ever breastfeed **[CHILDLAST/PRGTWINNAME*]**?	YES = 1<br>NO, NEVER BREASTFED = 2<br><br>DON'T KNOW = -8<br>REFUSED = -9	NO, DK, REFUSED → PREGNANT	N	Y	C

NO.	VARNAME	QUESTIONS	CODING CATEGORIES	SKIPS/FILTERS	INDICATOR	DHS/AIS	CORE
436	CHILDBFL ONG	For how long did you breastfeed **[CHILDLAST/PRGTWINNAME*]**?<br><br>ONLY ONE OPTION MAY BE SELECTED. FOR EXAMPLE, ANSWER ONLY IN WEEKS OR IN MONTHS.<br><br>CODE '00' IF LESS THAN 1 WEEK.	WEEKS ____<br>MONTHS ____<br>STILL BREASTFEEDING = 96<br>DON'T KNOW = -8<br>REFUSED = -9		N	N	C
437	CHILDMOR E	Thank you for the information regarding [CHILDLAST/PRGTWINNAME*].		IF PRGTWIN = 1 RETURN TO CHILDALIVE* FOR EACH VALUE OF PRGTWINNAM E*			

NO.	VARNAME	QUESTIONS	CODING CATEGORIES	SKIPS/FILTERS	INDICATOR	DHS/AIS	CORE
I will now ask about current pregnancies.							
438	PREGNAN T	Are you pregnant now?	YES = 1<br>NO = 2<br>DON'T KNOW/UNSURE = -8<br>REFUSED = -9	YES →SKIP TO END OF MODULE	N	Y	C

NO.	VARNAME	QUESTIONS	CODING CATEGORIES	SKIPS/FILTERS	INDICATOR	DHS/AIS	CORE
I will now ask you about family planning.							
439	AVOIDPREG	Are you or your partner currently doing something or using any method to delay or avoid getting pregnant?	YES = 1<br>NO = 2<br>DON'T KNOW = -8<br>REFUSED = -9	NO, DK, REFUSED→SKIP TO END OF MODULE	N	Y	C
440	CMETHOD	Which method are you or your partner using?<br><br>SELECT ALL THAT APPLY.	FEMALE STERILIZATION = A<br>MALE STERILIZATION = B<br>PILL = C<br>IUD/"COIL" = D<br>INJECTIONS = E<br>IMPLANT = F<br>CONDOM = G<br>FEMALE CONDOM = H<br>RHYTHM/NATURAL METHODS = I<br>WITHDRAWAL = J<br>NOT HAVING SEX = K<br>OTHER = X<br>SPECIFY: ____________________<br>DON'T KNOW = Y<br>REFUSED = Z		GF/HIV-P11	Y	C

MODULE 5: CHILDREN

THE HOUSEHOLD SCHEDULE NOTED THAT [NAME OF PARTICIPANT] WILL FILL OUT THE CHILDREN'S MODULE FOR [NUMBER OF CHILDREN].

I am going to ask you a number of questions about your child/children regarding their health and where they get their health services. I will begin with your youngest child. LIST OF CHILDREN < 14 ASSIGNED TO [INNAME]

NO.	VARNAME	QUESTIONS	CODING CATEGORIES	SKIPS/FILTERS	INDICATOR	DHS/AIS	CORE
	KIDINS1	Now I am going to ask you questions for **[CHILD*]**.			N	N	C
501	KIDAGEM/ KIDAGE[Y	How old is **[CHILD*]** in years?<br><br>IF [**CHILD***] IS LESS THAN 1 YEAR OLD, KEY 0 HERE AND KEY AGE IN MONTHS ON NEXT SCREEN.	YEARS ___<br>DON'T KNOW =-8<br>REFUSED = -9	>5, DK, REF →KIDGENDER<br>1-5 → KIDAGEF<br><br>AGE CANNOT BE GREATER THAN 14 YEARS.	N	N	C
502	KIDAGEM/ KIDAGEY	How old is **[CHILD*]** in months?	MONTHS ___<br>DON'T KNOW =-8<br>REFUSED = -9	ALL → KIDGENDER	N	N	C
503	KIDAGEF	You said that **[CHILD*]** was [KIDAGEY]. How many months over [KIDAGEY] is [**CHILD***].	MONTHS ____<br>DON'T KNOW = -8<br>REFUSED = -9				

NO.	VARNAME	QUESTIONS	CODING CATEGORIES	SKIPS/FILTERS	INDICATOR	DHS/AIS	CORE
504	KIDGENDER	Is **[CHILD*]** a boy or girl?	BOY = 1<br>GIRL = 2<br>DON'T KNOW = -8<br>REFUSED = -9		N	N	C
505	KIDENROLL	Is **[CHILD*]** enrolled in school?	YES = 1<br>NO, CURRENTLY NOT IN SCHOOL = 2<br>NO, TOO YOUNG TO BE IN SCHOOL = 3<br>DON'T KNOW = -8<br>REFUSED = -9	NO, CURR NOT IN SCHOOL→ KIDENRLSTYR<br>NO, TOO YOUNG, DK, REFUSED → KIDCRCM	N	N	C
506	KIDHIGHLVL	What is the highest level of school [**CHILD***] has attended: primary or secondary?	NURSERY = 1<br>PRIMARY = 2<br>SECONDARY = 3<br>DON'T KNOW = -8<br>REFUSED = -9	NURSERY,DK, REF → KIDCRCM	N	N	C
507	KIDCLASS	What standard/form/year is **[CHILD*]** in now?	STANDARD/FORM/YEAR _____<br>DON'T KNOW = -8<br>REFUSED = -9	ALL → KIDCRCM	N	N	C
508	KIDENRLSTYR	Was **[CHILD*]** enrolled in school during the previous school year?	YES = 1<br>NO = 2<br>DON'T KNOW = -8<br>REFUSED = -9	NO, DK, REF→KIDCRCM	N	N	C

NO.	VARNAME	QUESTIONS	CODING CATEGORIES	SKIPS/FILTERS	INDICATOR	DHS/AIS	CORE
509	KIDCLASSLSTYR	What standard/form/year was **[CHILD*]** during the previous school year?	STANDARD/FORM/YEAR _____<br>DON'T KNOW = -8<br>REFUSED = -9		N	N	C
510	KIDCRCM	Is [**CHILD***] circumcised?<br><br>Circumcision is the complete removal of the foreskin from the penis. If you feel comfortable, I can show you a picture of a completely circumcised penis.	YES = 1<br>NO = 2<br>DON'T KNOW = -8<br>REFUSED = -9	NO, DK, REF →KIDNOTCRC SKIP IF FEMALE CHILD.<br><br>ELECTRONIC AID IF REQUESTED.	N	N	C
511	KIDNOTCRC	Why is [**CHILD***] not circumcised?	DON'T KNOW WHERE TO GET CIRCUMCISED = A<br>HAD TO TRAVEL TOO FAR TO GET CIRCUMCISED = B<br>CHILD WAS AFRAID TO GET CIRCUMCISED = C<br>DO NOT HAVE A REASON TO CIRCUMCISE THE CHILD = D<br>WAITING UNTIL CHILD IS OLDER TO GET HIM CIRCUMCISED = E<br>RELIGIOUS REASONS = F<br>OTHER = G<br>SPECIFY: ____________________<br>DON'T KNOW = Y<br>REFUSED = Z				S

NO.	VARNAME	QUESTIONS	CODING CATEGORIES	SKIPS/FILTERS	INDICATOR	DHS/AIS	CORE
512	KIDCRCMPRT	Who circumcised [**CHILD***]?	DOCTOR, CLINICAL OFFICER, OR NURSE = 1<br>TRADITIONAL PRACTITIONER / CIRCUMCISER =2<br>MIDWIFE = 3<br>OTHER = 96<br>SPECIFY: ____________________<br>DON'T KNOW = -8<br>REFUSE TO ANSWER=-9	SKIP IF FEMALE CHILD.<br><br>ADAPT RESPONSES BASED ON COUNTRY CONTEXT	N	N	C
513	KIDHIVTESTEVR	Has **[CHILD*]** ever been tested for HIV?	YES = 1<br>NO = 2<br>DON'T KNOW = -8<br>REFUSED = -9	YES → KIDHIVTESTLASTM/KIDHIVTESTLASTY<br>DK, REFUSED → KIDVISTTBCLIN	N	N	C
514	KIDHIVTESTNEVERRSN	Why has **[CHILD*]** never been tested for HIV?<br><br>SELECT ALL THAT APPLY.	DON'T KNOW WHERE TO TEST = A<br>TEST COSTS TOO MUCH = B<br>TRANSPORT COSTS TOO MUCH = C<br>TOO FAR AWAY = D<br>AFRAID OTHERS WILL KNOW ABOUT TEST RESULTS = E<br>DON'T NEED TEST/LOW RISK = F<br>DID NOT RECEIVE PERMISSION FROM SPOUSE/FAMILY = G<br>AFRAID SPOUSE/PARTNER/FAMILY WILL KNOW RESULTS = H<br>DON'T WANT TO KNOW CHILD HAS HIV = I<br>CANNOT GET TREATMENT FOR HIV = J<br>TEST KITS NOT AVAILABLE = K<br>RELIGIOUS REASONS = L	ALL-> KIDVISTTBCLIN	N	N	C

NO.	VARNAME	QUESTIONS	CODING CATEGORIES	SKIPS/FILTERS	INDICATOR	DHS/AIS	CORE
			OTHER = X<br>SPECIFY: ____________________<br>DON'T KNOW = Y<br>REFUSED = Z				
515	KIDHIVTESTLASTM/KIDHIVTESTLASTY	What month and year was **[CHILD*]'s** last HIV test done?	MONTH ___ ___<br>DON'T KNOW MONTH = -8<br>REFUSED MONTH = -9<br><br>YEAR ___ ___ ___ ____<br>DON'T KNOW YEAR = -8<br>REFUSED YEAR = -9	DATE RESTRAINTS	N	N	C
516	KIDHIVLASTRESULT	What was [**CHILD***]'s last HIV test result?	POSITIVE = 1<br>NEGATIVE = 2<br>UNKNOWN/INDETERMINATE = 3<br>DID NOT RECEIVE RESULTS = 4<br>DON'T KNOW = -8<br>REFUSED = -9	IF NEG, UNK/INDET, DID NOT RECEIVE, DK, REFUSED→ KIDVISTTBCLIN	N	N	C
517	KIDLPOSM/KIDLPOSY	What was the month and year of [**CHILD***]'s first HIV positive test result? Please give your best guess.<br><br>This will be the very first HIV-positive test result that you have received.<br><br>PROBE TO VERIFY DATE.	MONTH ___ ___<br>DON'T KNOW MONTH = -8<br>REFUSED MONTH = -9<br><br>YEAR ___ ___ ___ ____<br>DON'T KNOW YEAR = -8<br>REFUSED YEAR = -9		N	N	C
518	KIDHIVCARE	Has [**CHILD***] ever received HIV medical care from a doctor, clinical officer or nurse?	YES = 1<br>NO = 2<br>DON'T KNOW = -8<br>REFUSED = -9	YES → KIDHIVCAREFIRSTM/KIDHIVCAREFIRSTY DK, REFUSED	N	N	C

NO.	VARNAME	QUESTIONS	CODING CATEGORIES	SKIPS/FILTERS	INDICATOR	DHS/AIS	CORE
				→ KIDCD4<br><br>ADAPT HEALTHCARE PROVIDER TERMS BASED ON LOCAL CONTEXT.			
519	KIDHIVCARENVR	What is the main reason why [**CHILD***] has never seen a doctor, clinical officer, or nurse for HIV medical care?	FACILITY IS TOO FAR AWAY = 1<br>I DON'T KNOW WHERE TO GET HIV MEDICAL CARE FOR CHILD = 2<br>COST OF CARE = 3<br>COST OF TRANSPORT = 4<br>I DON'T THINK CHILD NEEDS IT, HE/SHE IS NOT SICK = 5<br>I FEAR PEOPLE WILL KNOW THAT CHILD HAS HIV IF I TAKE HIM/HER TO A CLINIC = 6<br>RELIGIOUS REASONS = 7<br>CHILD IS TAKING TRADITIONAL MEDICINE = 8<br>OTHER = 96<br>SPECIFY: ____________________<br>DON'T KNOW = -8<br>REFUSED = -9	ALL→ KIDCD4<br><br>ADAPT RESPONSES TO LOCAL CONTEXT.<br><br>ADAPT HEALTHCARE PROVIDER TERMS BASED ON LOCAL CONTEXT.	N	N	C
520	KIDHIVCAREFIRSTM/KIDHIVCAREFIRSTY	What month and year did [**CHILD***] <u>first</u> see a doctor, clinical officer or nurse for HIV medical care?<br><br>PROBE TO VERIFY DATE.	MONTH __________<br>DON'T KNOW MONTH = -8<br>REFUSED MONTH= -9<br><br>YEAR ____________<br>DON'T KNOW YEAR =-8<br>REFUSED YEAR = -9		N	N	C

NO.	VARNAME	QUESTIONS	CODING CATEGORIES	SKIPS/FILTERS	INDICATOR	DHS/AIS	CORE
521	KIDHIVCARELASTM/KIDHIVCARELASTY	What month and year did [**CHILD***] <u>last</u> see a doctor, clinical officer or nurse for HIV medical care?	MONTH ___________<br>DON'T KNOW MONTH = -8<br>REFUSED MONTH= -9<br><br>YEAR _____________<br>DON'T KNOW YEAR =-8<br>REFUSED YEAR = -9	IF <7 MONTHS, DK, REFUSED, MISSING DATE → KIDCD4	N	N	C
522	KIDHIVNOT6RSN	What is the <u>main</u> reason for [**CHILD***] not seeing a doctor, clinical officer or nurse for HIV medical care for more than 6 months?	FACILITY IS TOO FAR AWAY = 1<br>I DON'T KNOW WHERE TO GET HIV MEDICAL CARE FOR CHILD = 2<br>COST OF CARE = 3<br>COST OF TRANSPORT = 4<br>I DON'T THINK CHILD NEEDS IT, HE/SHE IS NOT SICK = 5<br>I FEAR PEOPLE WILL KNOW THAT CHILD HAS HIV IF I TAKE HIM/HER TO A CLINIC = 6<br>RELIGIOUS REASONS = 7<br>CHILD IS TAKING TRADITIONAL MEDICINE = 8<br>NO APPOINTMENT SCHEDULED/DID NOT MISS MOST RECENT APPOINTMENT= 9<br>OTHER = 96<br>SPECIFY: ____________________<br>DON'T KNOW = -8<br>REFUSED = -9		N	N	C
523	KIDCD4	Has [**CHILD***] ever had a CD4 count test?<br>The CD4 count tells you how sick you are with HIV and if you need to take any HIV medications. All HIV infected people need to take ARVs.	YES = 1<br>NO = 2<br>DON'T KNOW = -8<br>REFUSED = -9	NO, DK, REFUSED → KIDARVS<br><br>NO, DK, REFUSED & NEVER IN HIV CARE →	N	N	C

NO.	VARNAME	QUESTIONS	CODING CATEGORIES	SKIPS/FILTERS	INDICATOR	DHS/AIS	CORE
				KIDVISTTBCLIN			
524	KIDCD4LASTM/KIDCD4LASTY	What month and year was [**CHILD***] last tested for his/her CD4 count?	MONTH ___ ___<br>DON'T KNOW MONTH = -8<br>REFUSED MONTH = -9<br><br>YEAR ___ ___ ___ ___<br>DON'T KNOW YEAR = -8<br>REFUSED YEAR = -9	SKIP TO KIDVISTTBCLIN IF NEVER IN HIV CARE.	N	N	C
525	KIDARVS	Has [**CHILD***] ever taken ARVs, that is, antiretroviral medications to treat his/her HIV infection?	YES = 1<br>NO = 2<br>DON'T KNOW = -8<br>REFUSED = -9	YES → KIDARVSFIRST7/KIDARVSFIRSTM<br>DK, REFUSED → KIDSEPTRIN<br><br>ELECTRONIC AID IF DON'T KNOW	N	N	C
526	KIDARVSNVRRSN	What is the main reason [**CHILD***] has never taken ARVs?	CHILD IS NOT ELIGIBLE FOR TREATMENT=1<br>HEALTH CARE PROVIDER DID NOT PRESCRIBE = 2<br>HIV MEDICINES NOT AVAILABLE = 3<br>DO NOT THINK CHILD NEEDS IT, HE/SHE IS NOT SICK = 4<br>COST OF MEDICATIONS = 5<br>COST OF TRANSPORT = 6<br>RELIGIOUS REASONS = 7<br>CHILD IS TAKING TRADITIONAL MEDICATIONS = 8	ALL→SKIP TO KIDSEPTRIN<br><br>ADAPT RESPONSES BASED ON COUNTRY CONTEXT.	N	N	C

NO.	VARNAME	QUESTIONS	CODING CATEGORIES	SKIPS/FILTERS	INDICATOR	DHS/AIS	CORE
			OTHER = 96<br>SPECIFY: ____________________<br>DON'T KNOW = -8<br>REFUSED = -9				
527	KIDARVSFIRST7/KIDARVSFIRSTM	What month and year did [**CHILD***] first start taking ARVs?<br>PROBE TO VERIFY DATE.	MONTH = ____<br>DON'T KNOW MONTH = -8<br>REFUSED MONTH = -9<br><br>YEAR = ____<br>DON'T KNOW YEAR = -8<br>REFUSED YEAR = -9		MER/TX_RET<br>GARPR/4.1<br>GF/HIV-I6	N	C
528	KIDARVSNOW	Is [**CHILD***] currently taking ARVs, that is, antiretroviral medications?<br><br>By currently, I mean that **[CHILD*]** may have missed some doses but [**CHILD*** is still taking ARVs.	YES = 1<br>NO = 2<br>DON'T KNOW = -8<br>REFUSED = -9	YES → KIDARVMISS30<br>DK, REFUSED → KIDSEPTRIN	N	N	C
529	KIDARVSNOTRSN	Can you tell me the main reason why [**CHILD***] is not currently taking ARVs?	I HAVE TROUBLE GIVING CHILD A TABLET EVERYDAY = 1<br>CHILD HAD SIDE EFFECTS/RASH = 2<br>FACILITY/PHARMACY TOO FAR AWAY TO GET MEDICATION REGULARLY = 3<br>COST OF MEDICATIONS = 4<br>COST OF TRANSPORT = 5<br>CHILD IS HEALTHY/, HE/SHE IS NOT SICK = 6<br>FACILITY WAS OUT OF STOCK = 7<br>RELIGIOUS REASONS= 8<br>**CHILD** IS TAKING TRADITIONAL MEDICATIONS = 9	ALL →KIDSEPTRIN<br><br>ADAPT RESPONSES BASED ON COUNTRY CONTEXT.	N	N	C

NO.	VARNAME	QUESTIONS	CODING CATEGORIES	SKIPS/FILTERS	INDICATOR	DHS/AIS	CORE
			OTHER =96<br>SPECIFY: ____________________<br>DON'T KNOW = -8<br>REFUSED = -9				
530	KIDARVMISS30	People sometimes forget to take all their ARVs every day. In the last 30 days, how many days has [**CHILD***] missed taking any ARV pills?<br>CODE '00' IF NONE.	DAYS ___ ___<br>DON'T KNOW = -8<br>REFUSED = -9		N	N	C
531	KIDSEPTRIN	Is [**CHILD***] currently taking Septrin, Dapsone or cotrimoxazole?<br><br>Septrin, Dapsone or cotrimoxazole is a medicine recommended for people with HIV, even if they have not started treatment for HIV. It helps prevent certain infections but it is not treatment for HIV.<br><br>By currently, I mean that [**CHILD***] may have missed some doses but is still taking Septrin, Dapsone or cotrimoxazole.	YES = 1<br>NO = 2<br>I DON'T KNOW WHAT IT IS = 3<br>REFUSED = -9	ADAPT TERM 'SEPTRIN' BASED ON COUNTRY CONTEXT.<br><br>ELECTRONIC AID IF DON'T KNOW	N	N	C
532	KIDVISTTBCLIN	Has [**CHILD***] ever visited a clinic for tuberculosis for TB diagnosis or treatment?	YES = 1<br>NO = 2<br>DON'T KNOW = -8<br>REFUSED = -9	NO, DK, REFUSED → SKIP TO END OF MODULE	N	N	C
533	KIDDIAGTB	Have you ever been told by a doctor, clinical officer or nurse that [**CHILD***] had TB?	YES = 1<br>NO=2<br>DON'T KNOW = -8<br>REFUSED = -9	NO,DK, REFUSED → SKIP TO END OF MODULE<br><br>ADAPT TERMS	N	N	C

NO.	VARNAME	QUESTIONS	CODING CATEGORIES	SKIPS/FILTERS	INDICATOR	DHS/AIS	CORE
				FOR HEALTHCARE PROVIDER TO COUNTRY CONTEXT.			
534	KIDTRTTB	Was [**CHILD***] ever treated for TB?	YES = 1<br>NO = 2<br>DON'T KNOW = -8<br>REFUSED = -9	NO, DK, REFUSED → SKIP TO END OF MODULE	N	N	C
535	KIDTRTTCURR	Is **[CHILD*]** currently on treatment for TB?	YES = 1<br>NO = 2<br>DON'T KNOW = -8<br>REFUSED = -9	NO, DK, REFUSED → KIDMORE	N	N	C
536	KIDTRTTB6MOTRT	The last time **[CHILD*]** was treated for TB, did [**CHILD***] complete at least 6 months of treatment?	YES = 1<br>NO = 2<br>DON'T KNOW = -8<br>REFUSED = -9		N	N	C
537	KIDMORE	Thank you for the information about **[CHILD*]**.<br><br>DOES THE RESPONDENT HAVE ANOTHER CHILD AGED 0-14 YEARS?	YES = 1<br>NO = 2	YES→RETURN TO KIDINS1	NA	NA	NA

MODULE 6: MALE CIRCUMCISION

NO.	VARNAME	QUESTIONS	CODING CATEGORIES	SKIPS/FILTERS	INDICATOR	DHS/AIS	CORE
I will be asking a few questions about circumcision. Circumcision is the removal of the foreskin from the penis. If you feel comfortable, I can show you a picture of circumcised penis.<br><br>DO NOT READ: SHOW AID				ELECTRONIC AID IF REQUESTED.<br><br>IF FEMALE → MCRISKR			
601	MCSTATUS	Some men are uncomfortable talking about circumcision but it is important for us to have this information. Some men are circumcised. Are you circumcised?	YES = 1<br>NO = 2<br>DON'T KNOW = -8<br>REFUSED=9	YES→MCAGE<br>NO → MCPLANS<br>DK, REF → MCRISKR	MER/VMMC_CIRC	Y(MODIFIED)	C
602	MCMETH	If so, were you medically circumcised, traditionally circumcised, or both?<br><br>By medically, we mean that your circumcision was done at the hospital or clinic, while traditional circumcision is done by a traditional doctor at the initiation school.	MEDICAL = 1<br>TRADITION = 2<br>BOTH = 3<br>DON'T KNOW = -8<br>REFUSED = -9	DK, REF → MCRISKR			Lesotho
603	MCPARCOM	Are you completely or partially circumcised?<br><br>In partial circumcision, a small part of the foreskin is removed from the penis while in complete circumcision, the entire foreskin is removed.<br><br>If you allow me, I can show you a picture that shows a partially and a completely circumcised penis.	COMPLETELY = 1<br>PARTIALLY = 2<br>DON'T KNOW = -8<br>REFUSED = -9	DK, REF → MCRISKR			Lesotho

NO.	VARNAME	QUESTIONS	CODING CATEGORIES	SKIPS/FILTERS	INDICATOR	DHS/AIS	CORE
604	MCAGE	How old were you when you were medically circumcised? Please give your best guess.<br><br>How old were you when you were traditionally circumcised? Please give your best guess.<br><br>IF LESS THAN ONE YEAR, CODE ‘00’.	MEDICAL:<br>COMPLETED AGE IN YEARS _____<br>DON'T KNOW = -8<br>REFUSED = -9<br><br>TRADITIONAL:<br>COMPLETED AGE IN YEARS _____<br>DON'T KNOW = -8<br>REFUSED= -9		N	Y	C
605	MCWHO	Who did the circumcision?	DOCTOR, CLINICAL OFFICER, OR NURSE= 1<br>RELIGIOUS LEADER = 2<br>TRADITIONAL PRACTITIONER, CIRCUMCISER/INITIATION SCHOOL PERSONNEL = 3<br>MIDWIFE = 4<br>FAMILY MEMBER/RELATIVE = 5<br>OTHER = 96<br>SPECIFY: ____________________<br>DON'T KNOW = -8<br>REFUSED = -9		N	Y	C
606	MCPLANS	Are you planning to get circumcised?	YES = 1<br>NO = 2<br>DON'T KNOW = -8<br>REFUSED= -9	YES → MCPLANTYPE<br>NO → MCNOPLANS<br>DK, REF → MCRISKR		N	C

NO.	VARNAME	QUESTIONS	CODING CATEGORIES	SKIPS/FILTERS	INDICATOR	DHS/AIS	CORE
607	MCPLANTYPE	What type of circumcision are you planning to get?	MEDICAL = 1<br>TRADITIONAL = 2<br>DON'T KNOW = -8<br>REFUSED = -9	ALL → MCRISKR			
608	MCNOPLANS	Why are you not planning to get circumcised?	DON'T KNOW WHERE TO GET CIRCUMCISED = A<br>HAD TO TRAVEL TOO FAR TO GET CIRCUMCISED = B<br>AFRAID TO GET CIRCUMCISED = C<br>DO NOT HAVE A REASON TO GET CIRCUMCISED = D<br>RELIGIOUS REASONS = E<br>NO REASON = F<br>OTHER = X<br>SPECIFY: ____________________<br><br>DON'T KNOW = Y<br>REFUSED = Z				Country added
609	MCRISKR	Does male circumcision alone reduce the risk, or chance, of a man getting HIV completely, somewhat or not at all?	PROTECTS COMPLETELY = 1<br>PROTECTS SOMEWHAT = 2<br>NOT AT ALL = 3<br>DON'T KNOW = 4<br>REFUSED = -9		N	N	S

MODULE 7: SEXUAL ACTIVITY

NO.	VARNAME	QUESTIONS	CODING CATEGORIES	SKIPS/FILTERS	INDICATOR	DHS/AIS	CORE
		In this part of the interview, I will be asking questions about your sexual relationships and practices. These questions will help us have a better understanding of how they may affect your life and risk for HIV.<br><br>Let me assure you again that your answers are completely confidential and will not be shared with anyone. If there are questions that you do not want to answer, we can go to the next question.					
701	FIRSTSXAGE	How old were you when you had vaginal sex for the very <u>first</u> time?<br><br>Vaginal sex is when a penis enters a vagina.<br><br>IF UNSURE, ASK THEM TO MAKE A BEST GUESS USING OTHER EVENTS IN THEIR LIFE THAT HAPPENED AROUND THE SAME TIME.<br><br>IF THE PARTICIPANT HAS NEVER HAD SEX, REFUSES, OR DOES NOT KNOW, SWIPE THROUGH WITHOUT ENTERING ANY RESPONSE. YOU WILL BE ABLE TO RECORD THESE RESPONSES ON THE SCREEN THAT FOLLOWS.	AGE IN YEARS _ _<br>NEVER HAD VAGINAL SEX = 96<br>DON'T KNOW = -8<br>REFUSED = -9	IF NEVER HAD VAGINAL SEX → SKIP TO ANSXEVER	GARPR/1.2<br>GF/HIV-O1	Y	C
702	FIRSTSEXCNDM	The first time you had vaginal sex was a condom used?	YES = 1<br>NO = 2<br>DON'T KNOW = -8<br>REFUSED = -9		N	N	S

NO.	VARNAME	QUESTIONS	CODING CATEGORIES	SKIPS/FILTERS	INDICATOR	DHS/AIS	CORE
703	FIRSTSEXWANT	The first time you had vaginal sex, was it because you wanted to or because you were forced?	WANTED TO = 1<br>FORCED = 2<br>DON'T KNOW = -8<br>REFUSED = -9		N	N	S
704	ANSXEVER	People have sex in different ways. Some have vaginal sex. Some have anal sex. Anal sex is when a penis enters a person's anus. Have you ever had anal sex?	YES = 1<br>NO = 2<br>DON'T KNOW = -8<br>REFUSED = -9	NO, DK, REFUSED<br><br>NEVER ANAL SEX __> FIRSTSEXPHYSF<br><br>NEVER VAGINAL OR ANAL SEX__> NEXT MODULE	N	N	S
705	FIRSTANALAGE	How old were you when you had anal sex for the very <u>first</u> time?	AGE IN YEARS _ _<br>DON'T KNOW = -8<br>REFUSED = -9		N	N	S
706	FIRSTANSEXCNDM	The first time you had anal sex was a condom used?	YES = 1<br>NO = 2<br>DON'T KNOW = -8<br>REFUSED = -9		N	N	S

NO.	VARNAME	QUESTIONS	CODING CATEGORIES	SKIPS/FILTERS	INDICATOR	DHS/AIS	CORE
707	FIRSTSEXANWANT	The first time you had anal sex, was it because you wanted to or because you were forced?	WANTED TO = 1<br>FORCED = 2<br>DON'T KNOW = -8<br>REFUSED = -9		N	N	S
708	FIRSTSEXPHYSF	How old was the person you first had vaginal or anal sex with? Please give your best guess.	AGE IN YEARS _____<br>DON'T KNOW = -8<br>REFUSED = -9		N	N	S
709	LIFETIMESEX	People often have sex with different people over their lifetime. In total, with how many different people have you had vaginal or anal sex in your lifetime? Please give your best guess.<br><br>IF NUMBER OF PARTNERS IS GREATER THAN 100, WRITE ' 100'.	NUMBER OF SEXUAL PARTNERS IN LIFETIME _ _ __<br><br>DON'T KNOW = -8<br>REFUSED = -9		N	Y	S
710	PART12MONUM	People often have sex with different partners over their lifetime. In total, with how many different people have you had vaginal or anal sex in the last 12 months?<br><br>IF NONE CODE '00'.<br><br>IF NUMBER OF PARTNERS IS GREATER THAN 100, WRITE ' 100'.	NUMBER OF SEXUAL PARTNERS IN LAST 12 MONTHS __ __ __<br><br>DON'T KNOW = -8<br>REFUSED = -9	IF 00 PARTNERS IN LAST 12 MONTHS → SKIP TO SELLSXEVER	GARPR/1.3 & 1.4<br>GF/HIV-O2 & HIV-O3	Y	C
		Now I would like to ask you some questions about the people you have had sex with in the last 12 months. Let me assure you again that your answers are completely confidential and will not be told to anyone. I will first ask you about your most recent partner.					

NO.	VARNAME	QUESTIONS	CODING CATEGORIES	SKIPS/FILTERS	INDICATOR	DHS/AIS	CORE
711	PARTLIVEW	Does [INITIALS] live in this household?	YES = 1<br>NO = 2	NO→PARTINIT	N	N	C
712	PARTHHLINE	DO NOT READ: HOUSEHOLD LINE NO. for [INITIALS]<br>CODE '0' IF NOT LISTED IN HOUSEHOLD ROSTER.	[LIST OF PERSONS FROM HOUSEHOLD]]<br>NOT LISTED IN HOUSEHOLD = 96	LISTED → PARTRELATION	N	N	C
713	PARTINIT	I would like to ask you for the initials of this person so I can keep track. They do not have to be the actual initials of this person.	INITIALS<br>____ ____		N	N	C
714	PARTRELATION	What is your relationship with (INITIALS)?	HUSBAND/WIFE = 1<br>LIVE-IN PARTNER = 2<br>PARTNER, NOT LIVING WITH RESPONDENT = 3<br>EX-SPOUSE/EX-PARTNER = 4<br>FRIEND/ACQUAINTANCE = 5<br>SEX WORKER = 6<br>SEX WORKER CLIENT =7<br>STRANGER = 8<br>OTHER = 96<br>SPECIFY: ______<br>DON'T KNOW = -8<br>REFUSED = -9		N	Y	C

NO.	VARNAME	QUESTIONS	CODING CATEGORIES	SKIPS/FILTERS	INDICATOR	DHS/AIS	CORE
715	PARTGEND	Is (INITIALS) male or female?	MALE = 1<br>FEMALE = 2<br>DON'T KNOW = -8<br>REFUSED = -9		N	N	C
716	PARTAGE	How old is **(INITIALS)**? Please give your best guess.	AGE IN YEARS ____<br>DON'T KNOW = -8<br>REFUSED = -9		N	N	C
717	PARTLASTCNDM	The <u>last</u> time you had sex with (INITIALS) was a condom used?	YES = 1<br>NO = 2<br>DON'T KNOW = -8<br>REFUSED = -9		GARPR/1.4<br>GF/HIV-O3	Y	C
718	PARTLASTSUP	Did you enter into a sexual relationship with (INITIALS) because (INITIALS) provided you with or you expected that (INITIALS) would provide you gifts, help you to pay for things, or help you in other ways?	YES = 1<br>NO = 2<br>DON'T KNOW = -8<br>REFUSED = -9	NO, DK, REFUSED → PARTAGAIN<br><br>SKIP IF SEX WORKER OR CLIENT	N	N	C
719	PARTLASTSUPREC	In the <u>last 12 months</u>, what have you received from (INITIALS)?<br><br>READ RESPONSES ALOUD.<br><br>SELECT ALL THAT APPLY.	DID NOT RECEIVE ANYTHING = A<br>MONEY = B<br>FOOD = C<br>SCHOOL FEES = D<br>EMPLOYMENT = E<br>GIFTS/FAVORS = F<br>TRANSPORT = G<br>SHELTER/RENT = H<br>PROTECTION = I<br>OTHER = X<br>SPECIFY: ______	SKIP IF SPOUSE, LIVE-IN PARTNER, SEX WORKER OR CLIENT	N	N	C

NO.	VARNAME	QUESTIONS	CODING CATEGORIES	SKIPS/FILTERS	INDICATOR	DHS/AIS	CORE
			DON'T KNOW = Y<br>REFUSED = Z				
720	PARTAGAIN	Do you expect to have sex with (**INITIALS**) again?	YES =1<br>NO =2<br>DON'T KNOW = -8<br>REFUSED = -9		N	N	C
721	PARTKNOWHIV	Does (INITIALS) know your HIV status? HIV status could mean you are HIV negative or HIV positive.	YES = 1<br>NO = 2<br>DON'T KNOW = -8<br>REFUSED = -9		N	N	C
722	PARTHIVSAT	What is the HIV status of (INITIALS)?<br><br>READ RESPONSES ALOUD	I THINK (INITIALS) IS POSITIVE = 1<br>(INTIALS) TOLD ME HE/SHE IS POSITIVE = 2<br>(INITIALS) IS POSITIVE, TESTED TOGETHER = 3<br>I THINK (INITIALS) IS NEGATIVE = 4<br>(INITIALS) TOLD ME HE/SHE IS NEGATIVE = 5<br>(INITIALS) IS NEGATIVE, TESTED TOGETHER=6<br>DON'T KNOW STATUS = 7<br>REFUSED = -9		N	N	C
723	PARTLASTSXTIME/PARTLASTSXUNITS	How long has it been since you last had sex with (INITIALS)?<br><br>DO NOT READ: IF LESS THAN ONE WEEK RECORD IN DAYS, IF LESS THAN ONE	DAYS _ _<br>WEEKS _ _<br>MONTHS _ _	DAYS_ _<br>WEEKS_ _<br>MONTHS _ _	DAYS _ _<br>WEEKS_ _<br>MONTHS _ _<br><br>DON'T KNOW		N	Y	S

NO.	VARNAME	QUESTIONS	CODING CATEGORIES			SKIPS/FILTERS	INDICATOR	DHS/AIS	CORE
		MONTH, RECORD IN WEEKS, OTHERWISE RECORD IN MONTHS.	DON'T KNOW = -8<br>REFUSED = -9	DON'T KNOW = -8<br>REFUSED = -9	= -8<br>REFUSED = -9				
724	PARTFIRSTSXTIME/PARTFIRSTSXUNITS	How long has it been since you <u>first</u> had sex with (INITIALS)?<br><br>DO NOT READ: IF LESS THAN ONE WEEK RECORD IN DAYS, IF LESS THAN ONE MONTH, RECORD IN WEEKS. IF LESS THAN ONE YEAR, RECORD, IN MONTHS, OTHERWISE RECORD IN YEARS.	DAYS _ _<br>WEEKS _ _<br>MONTHS _ _<br>YEARS _ _<br>DON'T KNOW = -8<br>REFUSED = -9	DAYS _ _<br>WEEKS _ _<br>MONTHS _ _<br>YEARS _ _<br>DON'T KNOW = -8<br>REFUSED = -9	DAYS _ _<br>WEEKS _ _<br>MONTHS _ _<br>YEARS _ _<br>DON'T KNOW = -8<br>REFUSED = -9		N	Y (MODIFIED)	S
725	PARTTIMES4WKS	In the last 4 weeks, what is the total number of times you had sex with (INITIALS)? By "times" we mean number of sex acts. For example, you could have sex 5 times with the same partner.<br><br>CODE '00' IF NONE.	NUMBER OF TIMES ____<br>DON'T KNOW = -8<br>REFUSED = -9	NUMBER OF TIMES ____<br>DON'T KNOW = -8<br>REFUSED = -9	NUMBER OF TIMES ____<br>DON'T KNOW = -8<br>REFUSED = -9		N	N	S
726	PARTCONDFREQVAG	In the last 12 months, how often did you use condoms with (INITIALS) when having vaginal sex? Was it always, most of the time, sometimes, rarely or never?	ALWAYS = 1<br>MOST OF THE TIME = 2<br>SOMETIMES = 3<br>RARELY = 4<br>NEVER = 5	ALWAYS = 1<br>MOST OF THE TIME = 2<br>SOMETIMES = 3<br>RARELY = 4<br>NEVER = 5	ALWAYS = 1<br>MOST OF THE TIME = 2<br>SOMETIMES = 3<br>RARELY = 4<br>NEVER = 5<br>NO VAGINAL SEX IN THE LAST 12		N	N	S

NO.	VARNAME	QUESTIONS	CODING CATEGORIES			SKIPS/FILTERS	INDICATOR	DHS/AIS	CORE
			NO VAGINAL SEX IN THE LAST 12 MONTHS = 3<br>DON'T KNOW = -8<br>REFUSED = -9<br><br>SKIP IF NEVER HAD VAGINAL SEX.	NO VAGINAL SEX IN THE LAST 12 MONTHS = 3<br>DON'T KNOW = -8<br>REFUSED = -9<br><br>SKIP IF NEVER HAD VAGINAL SEX.	MONTHS = 3<br>DON'T KNOW = -8<br>REFUSED = -9<br><br>SKIP IF NEVER HAD VAGINAL SEX.				
727	SXPREPS	DOES THE RESPONDENT HAVE ANOTHER PARTNER IN THE LAST 12 MONTHS?<br><br>I will now ask you about the person you have had sex with previous to (initials).	YES = 1<br>NO = 2			YES → PARTLIVEW	N	N	C

NO.	VARNAME	QUESTIONS	CODING CATEGORIES	SKIPS/FILTERS	INDICATOR	DHS/AIS	CORE
728	SELLSXEVER	Have you ever <u>had</u> sex for money and/or gifts?	YES = 1<br>NO = 2<br>DON'T KNOW = -8<br>REFUSED = -9	NO, DK, REFUSED → BUYSXEVER	N	N	S

NO.	VARNAME	QUESTIONS	CODING CATEGORIES	SKIPS/FILTERS	INDICATOR	DHS/AIS	CORE
729	SELLSX12 MO	In the last 12 months, have you <u>had</u> sex for money and/or gifts?	YES = 1<br>NO = 2<br>DON'T KNOW = -8<br>REFUSED = -9	NO, DK, REFUSED → BUYSXEVER	N	N	S
730	SELLSXCN DM	The last time you had sex for money and/or gifts, was a condom used?	YES = 1<br>NO = 2<br>DON'T KNOW = -8<br>REFUSED = -9		N	N	S
731	BUYSXEVE R	Have you <u>ever</u> paid money or given gifts for sex?	YES = 1<br>NO = 2<br>DON'T KNOW = -8<br>REFUSED = -9	NO, DK, REFUSED →<br><br>ENDOFMODUL E	N	N	S
732	BUYSX12M O	In the last 12 months, have you paid money or given gifts for sex?	YES = 1<br>NO = 2<br>DON'T KNOW = -8<br>REFUSED = -9	NO, DK, REFUSED →<br><br>ENDOFMODUL E	N	N	S
729	BUYSXCN DM	The last time you paid money or gave gifts for sex, was a condom used?	YES = 1<br>NO = 2<br>DON'T KNOW = -8<br>REFUSED = -9		N	N	S

MODULE 8: HIV KNOWLEDGE

NO.	VARNAME	QUESTIONS	CODING CATEGORIES	SKIPS/FILTERS	INDICATOR	DHS/AIS	CORE
Now I will ask you questions on your knowledge of HIV.				SELECT A SUBSET (I.E. HALF) OF PARTICIPANTS			
801	ONEPART NR	Can the risk of HIV transmission be reduced by having sex with only one uninfected partner who has no other partners?	YES = 1<br>NO = 2<br>DON'T KNOW = 3<br>REFUSED = -9		GARPR/1.1<br>GF/HIV-C-P1	Y	S
802	MOSQUITO	Can a person get HIV from mosquito bites?	YES = 1<br>NO = 2<br>DON'T KNOW = 3<br>REFUSED = -9		GARPR/1.1<br>GF/HIV-C-P1	Y	S
803	CONDOMS	Can a person reduce their risk of getting HIV by using a condom every time they have sex?	YES = 1<br>NO = 2<br>DON'T KNOW = 3<br>REFUSED = -9		GARPR/1.1<br>GF/HIV-C-P1	Y	S
804	SHAREFO OD	Can a person get HIV by sharing food with someone who has HIV?	YES = 1<br>NO = 2<br>DON'T KNOW = 3<br>REFUSED = -9		GARPR/1.1<br>GF/HIV-C-P1	Y	S
805	HEALTHYI NF	Can a healthy-looking person have HIV?	YES = 1<br>NO = 2<br>DON'T KNOW = 3<br>REFUSED = -9		GARPR/1.1<br>GF/HIV-C-P1	Y	S

NO.	VARNAME	QUESTIONS	CODING CATEGORIES	SKIPS/FILTERS	INDICATOR	DHS/AIS	CORE
Now I would like to ask you some questions about people's attitudes towards people living with HIV.							
806	BUYFOOD	Would you buy fresh vegetables from a shop keeper or vendor if you knew the person had HIV?	YES = 1<br>NO = 2<br>DON'T KNOW/NOT SURE/DEPENDS = -8<br>REFUSED = -9		GARPR 8.1	Y	S
807	KIDSSCHOOL	Do you think children living with HIV should be allowed to attend school with children who do not have HIV?	YES = 1<br>NO = 2<br>DON'T KNOW/NOT SURE/DEPENDS = -8<br>REFUSED = -9		GARPR 8.1	Y	S
808	FEARTEST	Do you think people hesitate to take an HIV test because they are afraid of how other people will react if the test result is positive for HIV?	YES = 1<br>NO = 2<br>DON'T KNOW/NOT SURE/DEPENDS = -8<br>REFUSED = -9		N	Y	S
809	TALKBAD	Do people talk badly about people who are living with HIV, or who are thought to be living with HIV?	YES = 1<br>NO = 2<br>DON'T KNOW/NOT SURE/DEPENDS = -8<br>REFUSED = -9		N	Y	S
810	RESPECT	Do people living with HIV, or thought to be living with HIV, lose the respect of other people?	YES = 1<br>NO = 2<br>DON'T KNOW/NOT SURE/DEPENDS = -8<br>REFUSED = -9		N	Y	S

NO.	VARNAME	QUESTIONS	CODING CATEGORIES	SKIPS/FILTERS	INDICATOR	DHS/AIS	CORE
811	SALIVA	Do you fear that you could get HIV if you come into contact with the saliva of a person living with HIV?	YES = 1<br>NO =2<br>ALREADY HAS HIV = 3<br>DON'T KNOW/NOT SURE/DEPENDS = -8<br>REFUSED = -9		N	Y(MODIFIED)	S
812	FAMSHAME	Do you agree or disagree with the following statement: I would be ashamed if someone in my family had HIV.	AGREE = 1<br>DISAGREE = 2<br>DON'T KNOW/NOT SURE/DEPENDS = -8<br>REFUSED = -9		N	Y	S

MODULE 9: HIV TESTING

NO.	VARNAME	QUESTIONS	CODING CATEGORIES	SKIPS/FILTERS	INDICATOR	DHS/AIS	CORE
I would now like to ask you some questions about HIV testing.							
901	HIVTSTEVER	Have you <u>ever</u> **tested** for HIV?	YES = 1<br>NO = 2<br>DON'T KNOW = -8<br>REFUSED = -9	YES → LIFETIMEHIVTEST<br>DK, REFUSED→SKIP TO END OF MODULE<br><br>CONSTRAINT IF REPORTED TESTING	GARPR/HTC-TST/GF HIV p.7	Y	C
902	HIVNOTTEST	Why have you never been tested for HIV?<br><br>SELECT ALL THAT APPLY.<br>READ ALL RESPONSE CATEGORIES	DON'T KNOW WHERE TO TEST = A<br>TEST COSTS TOO MUCH = B<br>TRANSPORT COSTS TOO MUCH = C<br>TOO FAR AWAY = D<br>AFRAID OTHERS WILL KNOW ABOUT TEST RESULTS = E<br>DON'T NEED TEST/LOW RISK = F<br>DID NOT RECEIVE PERMISSION FROM SPOUSE/FAMILY = G<br>AFRAID SPOUSE/PARTNER/FAMILY WILL KNOW RESULTS = H<br>DON'T WANT TO KNOW I HAVE HIV = I<br>CANNOT GET TREATMENT FOR HIV = J<br>TEST KITS NOT AVAILABLE = K<br>RELIGIOUS REASONS = L<br>OTHER = X		N	N	C

NO.	VARNAME	QUESTIONS	CODING CATEGORIES	SKIPS/FILTERS	INDICATOR	DHS/AIS	CORE
			SPECIFY: ____________________<br>DON'T KNOW = Y<br>REFUSED = Z				
903	LIFETIMEH IVTEST	In your lifetime, how many times have you been tested for HIV?	NUMBER OF TIMES TESTED FOR HIV<br>__ __<br><br>DON'T KNOW = -8<br>REFUSED = -9	CONSTRAINT SHOULD BE >=1	N	N	S
904	HIVTSLST M/HIVTSLS TY	What month and year was your last HIV test?	CALENDER MONTH _____ _____<br>DON'T KNOW MONTH= -8<br>REFUSED MONTH= -9<br><br>CALENDER YEAR ____<br>DON'T KNOW YEAR = -8<br>REFUSED YEAR= -9		GARPR/1.5 GF/HIV-C-P4	Y	C
905	HIVTESTL OCATION	Where was your last test done?	VCT FACILITY = 1<br>MOBILE VCT = 2<br>AT HOME = 3<br>HEALTH CLINIC/FACILITY = 4<br>HOSPITAL OUTPATIENT CLINIC = 5<br>TB CLINIC = 6<br>STI CLINIC = 7<br>HOSPITAL INPATIENT WARDS = 8<br>BLOOD DONATING CENTER = 9<br>ANC CLINIC = 10<br>OTHER = 96<br>SPECIFY: _____________________<br>DON'T KNOW = -8<br>REFUSED = -9				

NO.	VARNAME	QUESTIONS	CODING CATEGORIES	SKIPS/FILTERS	INDICATOR	DHS/AIS	CORE
906	HIVTSTRSLT	What was the result of that HIV test?	POSITIVE = 1<br>NEGATIVE = 2<br>UNCERTAIN/INDETERMINATE = 3<br>DID NOT RECEIVE THE RESULT = 4<br>DON'T KNOW = -8<br>REFUSED = -9	NEG, UNCERTAIN/IND, NO RESULT, DK, REF→SKIP TO END OF MODULE	GARPR/1.5<br>GF/HIV-C-P4	N	C
907	HIVTFPOSM/HIVTFPOSY	What was the month and year of your first HIV-positive test result? Please give your best guess.<br><br>This will be the very first HIV-positive test result that you have received.<br><br>DO NOT READ; PROBE TO VERIFY DATE.	CALENDER MONTH ___ ___<br>DON'T KNOW MONTH = -8<br>REFUSED MONTH = -9<br><br>CALENDER YEAR ___ ___ ___ ____<br>DON'T KNOW YEAR = -8<br>REFUSED YEAR = -9		N	N	C
908		Of the following people, to whom have you told that you are HIV positive?<br><br>CHECK ALL THAT APPLY. READ ALL RESPONSE CATEGORIES ALOUD.	NO ONE = A<br>SPOUSE/SEX PARTNER = B<br>DOCTOR = C<br>FRIEND = D<br>FAMILY MEMBER = E<br>OTHER = X<br>SPECIFY: ____________________<br>DON'T KNOW = Y<br>REFUSED = Z	SKIP TO NEXT QUESTION IF NO ONE, DK OR REFUSED.	N	N	C
Now I would like to ask you questions about your experiences with health care providers.				SKIP TO END OF MODULE IF NOT HIV POSITIVE.			

NO.	VARNAME	QUESTIONS	CODING CATEGORIES	SKIPS/FILTERS	INDICATOR	DHS/AIS	CORE
909	HIVSTATHIDE	In the last 12 months, when you sought health care in a facility where your HIV status is not known, did you feel you needed to hide your HIV status?	YES = 1<br>NO, NO NEED TO HIDE = 2<br>NO, DID NOT ATTEND HEALTH FACILITY IN LAST 12 MONTHS =3<br>DON'T KNOW = -8<br>REFUSED = -9	NO, DID NOT ATTEND HEALTH FACILITY→SKIP TO NEXT MODULE	N	N	C
910	HIVSTATDENIEDCARE	In the last 12 months, have you been denied health services including dental care, because of your HIV status?	YES = 1<br>NO = 2<br>NO ONE KNOWS MY STATUS = 3<br>DON'T KNOW = -8<br>REFUSED = -9		N	N	C

MODULE 10: HIV STATUS, CARE AND TREATMENT

NO.	VARNAME	QUESTIONS	CODING CATEGORIES	SKIPS/FILTERS	INDICATOR	DHS/AIS	CORE
Now I'm going to ask you more about your experience with HIV support, care and treatment.				SKIP TO NEXT MODULE IF NOT HIV POSITIVE			
1001	HIVCARE	After learning you had HIV, have you <u>ever</u> received HIV medical care from a doctor, clinical officer or nurse?	YES = 1<br>NO = 2<br>DON' T KNOW = -8<br>REFUSED = -9	YES → HIVCFM/HIVCFY<br>DK, REFUSED →SKIP TO END OF MODULE<br><br>ADAPT HEALTHCARE	N	N	C

NO.	VARNAME	QUESTIONS	CODING CATEGORIES	SKIPS/FILTERS	INDICATOR	DHS/AIS	CORE
				PROVIDER TERMS TO LOCAL CONTEXT.			
1002	HIVCNOTRSN	What is the main reason why you have never received HIV medical care from a doctor, clinical officer, or nurse?	FACILITY IS TOO FAR AWAY = 1<br>I DON'T KNOW WHERE TO GET HIV MEDICAL CARE = 2<br>COST OF CARE = 3<br>COST OF TRANSPORT = 4<br>I DO NOT NEED IT/I FEEL HEALTHY/NOT SICK = 5<br>I FEAR PEOPLE WILL KNOW THAT I HAVE HIV IF I GO TO A CLINIC = 6<br>RELIGIOUS REASONS = 7<br>I'M TAKING TRADITIONAL MEDICINE= 8<br>DO NOT TRUST THE STAFF/QUALITY OF CARE = 9<br>OTHER = 96<br>SPECIFY: ____________________<br>DON'T KNOW = -8<br>REFUSED = -9	SKIP TO CD4TESTEVER	N	N	C
1003	HIVCFM/HIVCFY	What month and year did you first see a doctor, clinical officer or nurse for HIV medical care?<br><br>DO NOT READ; PROBE TO VERIFY DATE.	CALENDER MONTH ___ ___<br>DON'T KNOW MONTH = -8<br>REFUSED MONTH = -9<br><br>CALENDER YEAR ___ ___ ___ ____<br>DON'T KNOW YEAR = -8<br>REFUSED YEAR = -9		N	N	C

NO.	VARNAME	QUESTIONS	CODING CATEGORIES	SKIPS/FILTERS	INDICATOR	DHS/AIS	CORE
1004	HIVCLM/HIVCLY	What month and year did you <u>last</u> see a doctor, clinical officer or nurse for HIV medical care?	CALENDER MONTH ___ ___<br>DON'T KNOW MONTH = -8<br>REFUSED MONTH= -9<br><br>CALENDER YEAR ___ ___ ___ ____<br>DON'T KNOW YEAR = -8<br>REFUSED = -9	IF <7 MONTHS, DK, REFUSED → CD4TESTEVER	N	N	C
1005	HIVCNOT6MO	What is the <u>main</u> reason for not seeing a doctor, clinical officer or nurse for HIV medical care for more than 6 months?	THE FACILITY IS TOO FAR AWAY = 1<br>I DON'T KNOW WHERE TO GET HIV MEDICAL CARE = 2<br>COST OF CARE = 3<br>COST OF TRANSPORT = 4<br>I DO NOT NEED IT/I FEEL HEALTHY/NOT SICK = 5<br>I FEAR PEOPLE WILL KNOW THAT I HAVE HIV IF I GO TO A CLINIC = 6<br>I'M TAKING TRADITIONAL MEDICINE= 7<br>RELIGIOUS REASONS = 8<br>NO APPOINTMENT SCHEDULED/DID NOT MISS MOST RECENT APPOINTMENT = 9<br>OTHER = 96<br>SPECIFY: ____________________<br>DON'T KNOW = -8<br>REFUSED = -9	ADAPT TIME BASED ON COUNTRY LOST-TO-FOLLOW-UP FOR PRE-ART HIV CARE.	N	N	C
1006	CD4TESTEVER	Have you ever had a CD4 count test?<br><br>The CD4 count tells you how sick you are with HIV and if you need to take ARVs or other HIV medications.	YES = 1<br>NO = 2<br>DON'T KNOW = -8<br>REFUSED = -9	NO, DK, REFUSED → ARVSTAKENEV<br><br>NO, DK, REFUSED &	N	N	C

NO.	VARNAME	QUESTIONS	CODING CATEGORIES	SKIPS/FILTERS	INDICATOR	DHS/AIS	CORE
				NEVER IN HIV CARE → SKIP TO END OF MODULE			
1007	CD4TTM/CD4TTY	What month and year were you last tested for your CD4 count?	CALENDAR MONTH ___ ___<br>DON'T KNOW MONTH = -8<br>REFUSED MONTH = -9<br><br>CALENDAR YEAR ___ ___ ___ ____<br>DON'T KNOW YEAR = -8<br>REFUSED YEAR = -9		N	N	C
1008	ARVSTAKENEV	Have you <u>ever</u> taken ARVs, that is, antiretroviral medications to treat HIV infection?	YES = 1<br>NO = 2<br>DON'T KNOW = -8<br>REFUSED = -9	YES→ ARVFTM/ARVFTY<br><br>DK, REFUSED → SKIP TO END OF MODULE	N	N	C
1009	ARVSNOTTAKE	What is the main reason you have never taken ARVs?	NOT ELIGIBLE FOR TREATMENT=1<br>HEALTH CARE PROVIDER DID NOT PRESCRIBE = 2<br>HIV MEDICINES NOT AVAILABLE = 3<br>I FEEL HEALTHY/NOT SICK = 3<br>COST OF MEDICATIONS = 4<br>COST OF TRANSPORT = 5<br>RELIGIOUS REASONS = 6<br>TAKING TRADITIONAL MEDICATIONS = 7<br>NOT ATTENDING HIV CLINIC = 8<br>OTHER = 96<br>SPECIFY: ____________________<br>DON'T KNOW = -8<br>REFUSED = -9	ALL→SKIP TO END OF MODULE<br><br>ADAPT RESPONSES BASED ON COUNTRY CONTEXT.	N	N	C

NO.	VARNAME	QUESTIONS	CODING CATEGORIES	SKIPS/FILTERS	INDICATOR	DHS/AIS	CORE
1010	ARVFTM/ARVFTY	What month and year did you <u>first</u> start taking ARVs?<br><br>DO NOT READ; PROBE TO VERIFY DATE.	CALENDER MONTH _____<br>DON'T KNOW MONTH = -8<br>REFUSED MONTH = -9<br><br>CALENDER YEAR _____<br>DON'T KNOW YEAR = -8<br>REFUSED YEAR = -9		MER/TX_RET<br>GARPR/4.1<br>GF/HIV-I6	N	C
1011	ARVSCURRENT	Are you <u>currently</u> taking ARVs, that is, antiretroviral medications?<br><br>By currently, I mean that you may have missed some doses but you are still taking ARVs.	YES = 1<br>NO=2<br>DON'T KNOW = -8<br>REFUSED = -9	YES→ARVSMISSDAYS<br>DK, REFUSED → SKIP TO END OF MODULE	MER/TX_CURR<br>GARPR/4.1<br>GF/HIV-T1	N	C
1012	ARVSNOTCURRSN	Can you tell me the <u>main</u> reason why you are <u>not</u> currently taking ARVs?	I HAVE TROUBLE TAKING A TABLET EVERYDAY = 1<br>I HAD SIDE EFFECTS = 2<br>FACILITY TOO FAR AWAY FOR ME TO GET MEDICINE REGULARLY = 3<br>COST OF MEDICATIONS = 4<br>COST OF TRANSPORT = 5<br>I FEEL HEALTHY/NOT SICK =6<br>FACILITY WAS OUT OF STOCK = 7<br>RELIGIOUS REASONS = 8<br>TAKING TRADITIONAL MEDICATIONS = 9<br>OTHER=96<br>SPECIFY: ____________________<br>DON' T KNOW = -8<br>REFUSED = -9	ALL → SKIP TO END OF MODULE<br><br>ADAPT RESPONSES BASED ON COUNTRY CONTEXT.	N	N	C

NO.	VARNAME	QUESTIONS	CODING CATEGORIES	SKIPS/FILTERS	INDICATOR	DHS/AIS	CORE
1013	ARVSMISSDAYS	People sometimes forget to take all of their ARVs every day. In the last 30 days, how many days have you missed taking any of your ARV pills?<br><br>DO NOT READ; CODE '00' IF NONE.	NUMBER OF DAYS ______<br>DON'T KNOW = -8<br>REFUSED = -9		N	N	C

MODULE 11: TUBERCULOSIS AND OTHER HEALTH ISSUES

NO.	VARNAME	QUESTIONS	CODING CATEGORIES	SKIPS/FILTERS	INDICATOR	DHS/AIS	CORE
Now we will ask you about tuberculosis or TB.							
1101	TBCLINVISIT	Have you ever visited a clinic for TB diagnosis or treatment?	YES = 1<br>NO=2<br>DON'T KNOW = -8<br>REFUSED = -9	NO, DK, REFUSED → SKIP TO END OF MODULE	N	N	C
1102	TBDIAGN	Have you ever been told by a doctor, clinical officer or nurse that you had TB?	YES = 1<br>NO=2<br>DON'T KNOW = -8<br>REFUSED = -9	NO, DK, REFUSED → SKIP TO END OF MODULE<br><br>ADAPT TERMS FOR HEALTHCARE PROVIDER TO COUNTRY CONTEXT.	N	N	C

NO.	VARNAME	QUESTIONS	CODING CATEGORIES	SKIPS/FILTERS	INDICATOR	DHS/AIS	CORE
1103	TBTREATED	Were you ever treated for TB?	YES = 1<br>NO = 2<br>DON'T KNOW = -8<br>REFUSED = -9	NO, DK, REFUSED → SKIP TO END OF MODULE	N	N	C
1104	TBTTREATCURR	Are you currently on treatment for TB?	YES = 1<br>NO = 2<br>DON'T KNOW = -8<br>REFUSED = -9	YES →SKIP TO END OF MODULE	N	N	C
1105	TBTREAT6MOFULL	The last time you were treated for TB, did you complete at least 6 months of treatment?	YES = 1<br>NO =2<br>DON'T KNOW = -8<br>REFUSED = -9		N	N	C

MODULE 12: GENDER NORMS

NO.	VARNAME	QUESTIONS	CODING CATEGORIES	SKIPS/FILTERS	INDICATOR	DHS/AIS	CORE
Now I would like to ask you question on attitudes and decision-making in your home.							
1201	HEALTHC	Who usually makes decisions about health care for yourself: you, your (spouse/partner), you and your (spouse/partner) together, or someone else?	I DO = 1<br>SPOUSE/PARTNER = 2<br>WE BOTH DO = 3<br>SOMEONE ELSE = 4<br>DON'T KNOW = -8<br>REFUSED = -9	SKIP IF NOT MARRIED/LIVING TOGETHER	N	Y	C
1202	MONEY	Who generally decides about how the money you receive is spent?	I DO = 1<br>SPOUSE/PARTNER = 2	SKIP IF NOT MARRIED/LIVI	N	Y	C

NO.	VARNAME	QUESTIONS	CODING CATEGORIES	SKIPS/FILTERS	INDICATOR	DHS/AIS	CORE
		READ ANSWER CHOICES ALOUD.	WE BOTH DO = 3<br>SOMEONE ELSE = 4<br>DON'T KNOW = -8<br>REFUSED = -9	NG TOGETHER			
1203	HHPURCH	Who usually makes decisions about making major household purchases: you, your (spouse/partner), you and your (spouse/partner) together or someone else?	I DO = 1<br>SPOUSE/PARTNER = 2<br>WE BOTH DO = 3<br>SOMEONE ELSE = 4<br>DON'T KNOW = -8<br>REFUSED = -9	SKIP IF NOT MARRIED/LIVI NG TOGETHER	N	Y	S
1204	OKGOOUT	Do you believe it is right for a man to hit or beat his wife if she goes out without telling him?	YES = 1<br>NO = 2<br>DON'T KNOW = -8<br>REFUSED = -9		N	N	S
1205	OKVIOLEN CE	Do you believe a person should tolerate violence from a spouse/partner to keep the family together?	YES = 1<br>NO = 2<br>DON'T KNOW = -8<br>REFUSED = -9		N	N	S
1206	DECDSX	Who should decide when to have sex—only the man, only the woman, or the man and woman together?	MEN ONLY = 1<br>WOMEN ONLY = 2<br>TOGETHER = 3<br>DON'T KNOW = -8<br>REFUSED = -9		N	N	S
1207	CNDMSEX	Do you believe women who carry condoms have sex with a lot of men?	YES = 1<br>NO = 2<br>DON'T KNOW = -8<br>REFUSED = -9		N	N	S

MODULE 13: VIOLENCE

NO.	VARNAME	QUESTIONS	CODING CATEGORIES	SKIPS/FILTERS	INDICATOR	DHS/AIS	CORE
You have been selected to be asked questions on other important aspects of a person's life. I know that some of these questions are very personal. However, your answers are important for helping to understand the condition of men and women in Lesotho. You will be the only person in your household who is asked these questions. Let me assure you that your answers are completely confidential and will not be told to anyone and no one in your household will know that you were asked these questions.<br><br>By sex, I mean vaginal, anal, oral sex or the insertion of an object into your vagina or anus. Vaginal sex is when a penis enters a vagina. Anal sex is when a penis enters an anus (butt). Oral sex is when a partner puts his/her mouth on his/her partner's penis or vagina.				SELECT ONLY 1 PERSON PER HOUSEHOLD.<br><br>SHOULD ALWAYS BE LAST MODULE IN QUESTIONNAIRE.			
1301	RSSXTIMES	How many times in your life has someone <u>pressured</u> you to have sex through harassment, threats and tricks and did succeed?<br><br>Being pressured can include being worn down by someone who repeatedly asks for sex, feeling pressured by being lied to, being told promises that were untrue, having someone threaten to end a relationship or spread rumors or sexual pressure due to someone using their influence or authority.<br><br>CODE '00' IF NONE.	NUMBER OF TIMES____<br>DON'T KNOW = -8<br>REFUSED = -9	NONE, DK, REFUSED→ END OF MODULE	N	N	S

NO.	VARNAME	QUESTIONS	CODING CATEGORIES	SKIPS/FILTERS	INDICATOR	DHS/AIS	CORE
1302	RSSXAGE	How old were you the first time someone pressured you to have sex and did succeed?	AGE IN YEARS ____<br>DON'T KNOW = -8<br>REFUSED = -9		N	N	S
1303	RSSX12MO	In the last 12 months, did someone pressure you to have sex and did succeed?	YES = 1<br>NO = 2<br>DON'T KNOW = -8<br>REFUSED = -9	NO, DK, REFUSED → RCSXTIMES	N	N	S
1304	RSSXLSTREL	What was this person's relationship to you? If it was more than one person, what was your relationship with the person you knew the best?	BOYFRIEND/GIRLFRIEND/LIVE-IN PARTNER/SPOUSE = 1<br>EX-BOYFRIEND/GIRLFRIEND/PARTNER/SPOUSE = 2<br>RELATIVE/FAMILY MEMBER = 3<br>CLASSMATE/SCHOOLMATE = 4<br>TEACHER = 5<br>POLICE/SECURITY OFFICER/MILITARY= 6<br>EMPLOYER = 7<br>NEIGHBOR = 8<br>COMMUNITY/ RELIGIOUS LEADER = 9<br>FRIEND = 10<br>STRANGER = 11<br>OTHER = 96<br>SPECIFY: ____________________<br>DON'T KNOW = -8<br>REFUSED = -9	SKIP IF ONLY HAPPENED ONCE.	N	N	S
1305	RCSXTIMES	How many times in your life have you been physically forced to have sex?<br><br>CODE '00' IF NONE.	NUMBER OF TIMES ____<br>DON'T KNOW = -8<br>REFUSED = -9	NONE, DK, REFUSED→ RCSX12MO	N	N	S
1306	RCSXAGE	How old were you the first time someone physically forced you to have sex?	AGE IN YEARS _____<br>DON'T KNOW = -8		N	N	S

NO.	VARNAME	QUESTIONS	CODING CATEGORIES	SKIPS/FILTERS	INDICATOR	DHS/AIS	CORE
			REFUSED = -9				
1307	RCSX12MO	In the last 12 months, did someone physically force you to have sex?	YES = 1<br>NO = 2<br>DON'T KNOW = -8<br>REFUSED = -9	NO, DK, REFUSED → RCSX12MOPT	N	N	S
1308	RCSXLSTREL	What was this person's relationship to you? If it was more than one person, what was the relationship with the person you knew the best?	BOYFRIEND/GIRLFRIEND/LIVE-IN PARTNER/SPOUSE = 1<br>EX-BOYFRIEND/GIRLFRIEND/PARTNER/SPOUSE = 2<br>RELATIVE/FAMILY MEMBER = 3<br>CLASSMATE/SCHOOLMATE = 4<br>TEACHER = 5<br>POLICE/SECURITY OFFICER/MILITARY= 6<br>EMPLOYER = 7<br>NEIGHBOR = 8<br>COMMUNITY/ RELIGIOUS LEADER = 9<br>FRIEND = 10<br>STRANGER = 11<br>OTHER = 96<br>SPECIFY: ____________________<br>DON'T KNOW = -8<br>REFUSED = -9	SKIP IF ONLY HAPPENED ONCE.	N	N	S
1309	RCSX12MOPT	In the last 12 months, did a partner physically force you to have sex?<br><br>By partner, I mean a live-in partner whether or not you were married at the time.	YES =1<br>NO, DID NOT FORCE = 2<br>NO, DID NOT HAVE A LIVE-IN PARTNER IN THE LAST 12 MONTHS = 3<br>DON'T KNOW = -8<br>REFUSED = -9		GARPR 7.1	N	S
1310	NWNTSEEK	After any of these unwanted sexual experiences, did you try to seek professional help or services from any of the following?	I DID NOT TRY TO SEEK HELP = A<br>HEALTHCARE PROFESSIONAL = B<br>POLICE OR OTHER SECURITY	DID NOT TRY TO SEEK HELP → END OF MODULE	N	N	S

NO.	VARNAME	QUESTIONS	CODING CATEGORIES	SKIPS/FILTERS	INDICATOR	DHS/AIS	CORE
		SELECT ALL THAT APPLY.	PERSONNEL = C<br>SOCIAL WORKER, COUNSELOR OR NON-GOVERNMENTAL ORGANIZATION = D<br>RELIGIOUS LEADER = E<br>OTHER = X<br>SPECIFY: ____________________<br>DON'T KNOW = Y<br>REFUSED = Z	SKIP IF NEVER EXPERIENCED SEXUAL VIOLENCE.			
1311	NWNTSXNOHLP	What was the main reason that you did not try to seek professional help or services?	DID NOT KNOW SERVICES WERE AVAILABLE = 1<br>SERVICES NOT AVAILABLE = 2<br>AFRAID OF GETTING IN TROUBLE = 3<br>ASHAMED FOR SELF/FAMILY = 4<br>COULD NOT AFFORD SERVICES = 5<br>DID NOT THINK IT WAS A PROBLEM = 6<br>FELT IT WAS MY FAULT = 7<br>AFRAID OF BEING ABANDONED = 8<br>DID NOT NEED/WANT SERVICES = 9<br>AFRAID OF MAKING SITUATION WORSE = 10<br>OTHER = 96<br>SPECIFY: ____________________<br>DON'T KNOW = -8<br>REFUSED = -9	SKIP IF NEVER EXPERIENCED SEXUAL VIOLENCE.	N	N	S
	VIOLMSG	Thank you for sharing your personal experiences with me. I know it may have been difficult for you to talk about your experiences with me. If you would like to talk further about these experiences, I can refer you to a place that can provide you with help.<br><br>PROVIDE PARTICIPANT WITH LIST OF ORGANIZATIONS.		SKIP IF NEVER EXPERIENCED SEXUAL OR PHYSICAL VIOLENCE.			

		You mentioned earlier that you have sold sex for money. Thank you for sharing your personal experiences with me. If you want to talk further about these experiences, I can refer you to a place that can provide you with help.<br><br>FILL OUT REFERRAL FORM FOR CHILDREN IDENTIFIED AS TRAFFICKED MINORS. FILL OUT SUMMARY OF REFERRED TRAFFICKED MINORS. PROVIDE PARTICIPANT WITH LIST OF ORGANIZATIONS, IF NOT ALREADY GIVEN.		SKIP IF >18 YEARS OLD<br><br>SKIP IF NEVER SOLD SEX	NA	NA	NA
		Thank you for taking the time to take part in this survey. Your responses will be very helpful to the Ministry of Health to better understand how to improve health programs in the country.<br><br>PROVIDE PARTICIPANT WITH LIST OF ORGANIZATIONS, IF NOT ALREADY GIVEN.			NA	NA	NA

COMMENTS FROM INTERVIEWER:

____

____

____

____
