# Supplementary material for: Association between severe drought and HIV prevention and care behaviors in Lesotho: A population-based survey 2016–2017
Source: PLoS Med. 2019 Jan 14;16(1):e1002727. doi: 10.1371/journal.pmed.1002727 (PMC6331084; doi:10.1371/journal.pmed.1002727)
Supplement: S1 Analysis Plan — (DOCX) [file pmed.1002727.s002.docx]

**S1 Analysis Plan**

*Please complete a proposal form for each proposed abstract/manuscript and submit it to the MC-SPC at ICAP. The following items are requested for each publication proposal. The starred items (*) are required.*

**Proposal submitted by (Name, Institutional Affiliation)*:**

| Low, A (ICAP-NY) |
| --- |

**Date of proposal submission to SPC*:**

| 6.22.2017 |
| --- |

**Email address of lead author*:**

| Al3546@cumc.columbia.edu |
| --- |

**Working title*:**

| Impact of recent drought on HIV and other indicators in Southern Africa |
| --- |

**Research Type*:**

|  | Tick if yes |
| --- | --- |
| Data-driven/ Results | X |
| Methods or Process description |  |

**Research question(s)***:

| 1. Are severe local rainfall shocks associated with HIV infection, increased risk behavior, migration and disruptions in the continuum in care in Southern Africa? |
| --- |

**Proposed lead analyst (Name, Institutional Affiliation)*:**

| Andrea Low, ICAP At Columbia |
| --- |

**Proposed methods for data-driven manuscripts (leave this section blank for non-data-driven projects):**

**Data sources for analysis:**

| *Please tick for each data source:* | *First Report Dataset* | *Final Report Dataset* |
| --- | --- | --- |
| Household dataset | X | X |
| Individual dataset | X | X |
| Individual biomarker/blood draw dataset | X | X |

**Names of country PHIA surveys to be included*:**

| Zimbabwe, Malawi, Zambia, Swaziland and Lesotho |
| --- |

**PHIA objectives addressed in this publication:**

| Factors contributing to HIV risk |
| --- |

**Sample and inclusion/exclusion criteria*** (e.g. 10-14 year olds, children < 5 years of age, pre-ART patients, etc.)**:**

| Adults >=15 years |
| --- |

**Main outcome variables and definitions*** (e.g., CD4 count):

| HIV prevalence, incidence, self-report ART, measured ARVs where available, CD4, risk behavior, VLS, HH size or structure |
| --- |

**Other variables of interest** (e.g., age, sex, education level):

| Age, sex, education level, marital status, age at marriage (female), intergenerational sex, transactional or commercial sex, wealth quintile |
| --- |

**Statistical methods for analyses*** (e.g., Chi-square tests, linear regression)**:**

| Regression analysis of odds of HIV prevalence and incidence in areas of low (<15% of normal distribution) rainfall (or other definition of rainfall shock) compared to areas with normal rainfall, with comparison of impact in rural vs urban areas and areas of high and low prevalence of HIV. |
| --- |

**Please briefly describe the abstract/ manuscript*:**

| Southern Africa recently experienced the most severe drought to hit in 35 years, affecting roughly 18 million people, in countries such as Zimbabwe, Malawi and Lesotho, particularly in rural areas. Previous studies have suggested that income shocks attributable to decreases in local rainfall could be responsible for up to 20% of variation in HIV prevalence across sub-Saharan Africa. The concurrence and scope of the PHIAs taking place from 2015-2017 in countries in Southern Africa allow for a detailed analysis of the association between rainfall shortages and HIV prevalence. Using geospatial data on HIV cases paired with precipitation measurements collected during this same period, an analysis can be done on the association between rainfall and a number of outcomes:  1. HIV prevalence- this will be analysed as a total, and disaggregated into HIV in vulnerable populations, namely AGYW.  2. HIV incidence (acknowledging likely inadequate sample size)  3. HIV treatment measurements (self-report enrolment in care, ART use, CD4, VLS, measured ARVs).  4. HH socio-economic status as reported in HH interview and classified as wealth quintile, educational attainment of children <18  5. Migration (Lesotho only)  6. Risk behaviours on causal pathway- transactional or commercial sex, condom use, age-mixing |
| --- |

**Additional comments:**

| To determine rainfall ‘shock’ in terms of agricultural productivity, data from the WFP will be used to generate an income shock variable as a binary indicator for whether an EA has experienced a shock crop year or not, using a number of different definitions of shock, based on the literature.  This analysis benefits from a multicountry pooling of data due to limited sample size in a country analysis, and by allowing a cross-country comparison of impact. |
| --- |
