# Supplementary material for: Association between severe drought and HIV prevention and care behaviors in Lesotho: A population-based survey 2016–2017
Source: PLoS Med. 2019 Jan 14;16(1):e1002727. doi: 10.1371/journal.pmed.1002727 (PMC6331084; doi:10.1371/journal.pmed.1002727)
Supplement: S1 STROBE — (DOC) [file pmed.1002727.s003.doc]

STROBE Statement—Checklist of items that should be included in reports of ***cross-sectional studies***

|  | Item No | Recommendation |
| --- | --- | --- |
| **Title and abstract** | 1 | *(*a) Indicate the study’s design with a commonly used term in the title or the abstract  [Title page, 1^st^ line] *Association between severe drought and HIV prevention and care behaviors in Lesotho: a population-based survey* |
|  |  | (*b*) Provide in the abstract an informative and balanced summary of what was done and what was found  [Abstract, paragraph 2 ]  *Methods and Findings: LePHIA selected a nationally representative sample between November 2016 and May 2017. All adults aged 15-59 years in randomly selected households were invited to complete an interview and HIV testing, with one woman per household eligible to answer questions on their experience of sexual violence. Deviations in rainfall for May 2014-June 2016 were estimated using precipitation data from Climate Hazards Group InfraRed Precipitation with Station Data (CHIRPS), with drought defined as <15% of the average rainfall from 1981-2016. The association between drought and risk behaviors as well as HIV-related outcomes was assessed using logistic regression, incorporating complex survey weights. Analyses were stratified by age, sex and geography (urban vs rural). All of Lesotho suffered from reduced rainfall, with regions receiving 1% to 31% of their historical rainfall. Of the 12887 interviewed participants, 93.5% (12052) lived in areas that experienced drought, with the majority in rural areas (7281 vs 4771 in urban areas). Of the 835 adults living in areas without drought, 520 were in rural areas, and 315 in urban. Among 15-19 year old females, living in a rural drought area was associated with early sexual debut (odds ratio [OR] 3.11, 95% CI 1.43-6.74, p=0.004), and higher HIV prevalence (OR 2.77, 95% CI 1.19-6.47, p=0.02). It was also associated with lower educational attainment in rural females ages 15-24 (OR 0.44, 95% CI 0.25-0.78, p=0.005). Multivariable analysis adjusting for household wealth and sexual behavior showed that experiencing drought increased the odds of HIV infection among 15-24 year old females (adjusted odds ratio [aOR] 1.80 (0.96-3.39) p=0.07), although this was not statistically significant. Migration was associated with a two-fold higher odds of HIV infection in young people (aOR 2.06, 95% CI 1.25-3.40, p=0.006).* |
| Introduction | | |
| Background/rationale | 2 | Explain the scientific background and rationale for the investigation being reported  [Introduction, paragraph 1] *The impact of climate change on human health is becoming increasingly evident. Aside from changes in infectious disease transmission directly related to disturbances favoring multiplication of disease vectors, periods of climate extremes are often associated with changes in behavior as people struggle to survive in the face of loss of agricultural production [1, 2]. As people, particularly women, address their food insecurity, they may be less likely to take steps to protect themselves from HIV infection [3, 4], and studies have documented increases in HIV infections during drought-related famine periods in Africa [5, 6]. A previous study of 21 Demographic and Health Surveys (DHS) across 19 countries in sub-Saharan Africa from 2003-2009 demonstrated that recurrent rainfall shocks were likely responsible for approximately 11% of HIV infections due to negative income shocks, particularly in high prevalence countries and in predominantly agrarian societies [6]. There is also concern that food insecurity could lead to decreased access to antiretrovirals (ARVs) due to economic constraints, or decreased adherence or absorption of ARVs [7-9], with a subsequent increase in community viral load, drug resistance, and HIV transmission [10-13]. Despite the increased frequency and severity of droughts in the Sahel and Southern Africa, few countries’ climate change adaptation policies currently include any intensified efforts for HIV prevention during climate-related events [14].*  *Southern Africa experienced two years of an El Nino-induced regional drought during the growing seasons of 2014-2015, including during key stages of crop development, leading to food shortages in 2016 and increased food costs for almost 40 million people in the region [15]. In Lesotho, where 55% of the population grow their own food, most people survive on rain-fed subsistence farming [16]. The drought led to a 67% reduction in maize production, with almost 25% of the population requiring emergency food assistance by August of 2016 [16]. Lesotho also has a long tradition of labor migration to South Africa, and, during the drought it was expected that migration would increase, particularly from predominantly rural districts [17]. Furthermore, Lesotho is a country with a hyper-endemic HIV epidemic, with prevalence above 25% in the adult population, and therefore at greater risk of disruption to any improvements in epidemic control [3, 18].* |
| Objectives | 3 | State specific objectives, including any prespecified hypotheses  [Introduction, paragraph 3] *This study used LePHIA data to assess whether people living in areas most severely affected by the drought had higher HIV prevalence, or changes in risk behaviors, and whether there was any difference in the continuum of care among people living with HIV (PLHIV). The analysis additionally disaggregates by age band to examine the outcomes in both youth and older people [18][19].* |
| Methods | | |
| Study design | 4 | Present key elements of study design early in the paper  [Methods, paragraph 1] *LePHIA employed a two-stage sampling design to select a nationally representative sample of adults and children aged 0-59 years in 418 enumeration areas (EA) across all 10 districts. The sample size was powered on a relative standard error of 30% for incidence of HIV in adolescent girls and young women (AGYW). Consenting heads of household completed a household questionnaire, including a roster of all household members who resided in or had slept in the household the previous night. These individuals were then consented to a questionnaire on socio-demographic and behavioral factors, and to home-based HIV testing. A guardian or parent provided permission to approach 10-17 year olds who were then asked for assent for all procedures. The adult questionnaire was administered to participants aged 15-59.* |
| Setting | 5 | Describe the setting, locations, and relevant dates, including periods of recruitment, exposure, follow-up, and data collection  [Introduction, paragraph 3] *The Lesotho Population-based HIV Impact Assessment (LePHIA) was a national survey conducted from November 2016 to May 2017*.  [Methods, paragraph 1] *LePHIA employed a two-stage sampling design to select a nationally representative sample of adults and children aged 0-59 years in 418 enumeration areas (EA) across all 10 districts. The sample size was powered on a relative standard error of 30% for incidence of HIV in adolescent girls and young women (AGYW). Consenting heads of household completed a household questionnaire, including a roster of all household members who resided in or had slept in the household the previous night. These individuals were then consented to a questionnaire on socio-demographic and behavioral factors, and to home-based HIV testing. A guardian or parent provided permission to approach 10-17 year olds who were then asked for assent for all procedures. The adult questionnaire was administered to participants aged 15-59.* |
| Participants | 6 | (*a*) Give the eligibility criteria, and the sources and methods of selection of participants  [Methods, paragraph 1] *A guardian or parent provided permission to approach 10-17 year olds who were then asked for assent for all procedures. The adult questionnaire was administered to participants aged 15-59. Written informed consent was documented at each stage via electronic signature.* |
| Variables | 7 | Clearly define all outcomes, exposures, predictors, potential confounders, and effect modifiers. Give diagnostic criteria, if applicable  [Methods, paragraph 6] *The effect of the drought was examined on recent behavior (over the past 12 months), HIV prevalence, and the continuum of HIV care at the individual level, using weighted data for all analyses, per an a priori analysis plan (Supplementary materials S1). Commercial and forced sex were lifetime measures, due to the smaller numbers of respondents*  [Methods, paragraph 7] *We examined the association with behaviors associated with HIV acquisition, such as recent condom use, commercial sex [21], and migration [22], and in young people for whom the drought would be most likely to affect recent transitions, educational attainment [24], transactional and intergenerational sex (sexual partner who was 10 or more years older), and early sex and marriage in 15-19 year olds [23, 24]. Transactional sex was defined as non-marital, non-commercial sex entered into on the assumption of material benefits [25], and high-risk sex as reporting sex without a condom with someone suspected or known to be HIV-positive. Educational attainment was defined as whether they had attended secondary school or greater, and did not require completion. Food insecurity was defined as any 24-hour period without food to eat because of lack of resources in the past four weeks. To assess the impact on the treatment cascade, we assessed the association of drought with awareness of HIV status, reported ART use, and VLS, defined as HIV RNA<1000 copies/ml in PLHIV.* |
| Data sources/ measurement | 8 | For each variable of interest, give sources of data and details of methods of assessment (measurement). Describe comparability of assessment methods if there is more than one group  [Methods, paragraph 2] *Survey staff administered the household and the adult questionnaires during a face-to-face interview with participants using Google Nexus 9 tablets. The household questionnaire collected data on household assets and access to food. The adult questionnaire included questions on lifetime and recent sexual behaviors, as well as questions on the HIV continuum of care for those who reported being HIV positive.*  [Methods, paragraph 3] *Rapid HIV testing was conducted using point of care (POC) tests, Determine HIV-1/2 Rapid Test (Alere), confirmed with Uni-Gold HIV Test (Trinity Biotech), as per the national algorithm. Laboratory verification of all HIV-positive results was done using the Geenius HIV-1/2 supplemental assay (Bio-Rad). Viral load testing was done preferentially on plasma or on dried blood spots if necessary (DBS), at a central lab on an automated platform.*  [Methods, paragraph 4] *Drought was quantified using precipitation estimates from the Climate Hazards Group InfraRed Precipitation with Station data (CHIRPS) which blends a variety of satellite imagery with interpolated weather station data to create gridded rainfall estimates at dekadal time-step and 0.05° resolution, or approximately 30 km2 [18].* |
| Bias | 9 | Describe any efforts to address potential sources of bias  [Methods, paragraph 2] *Only one female participant aged 15 or older in each household was randomly selected to answer questions about experiences with sexual violence, to mask the nature of the questions to other members of the household.*  [Methods, paragraph 6] *Design weights were calculated based on sampling design, including probability of household selection, and adjusted for non-response at the household, individual and biomarker level using the Chi-Squared Automatic Interactor Detector (SI-CHAID) software (Statistical Innovations); this was stratified by urban or rural residence, age group, and region, with peri-urban populations grouped with rural as per the Lesotho Bureau of Statistics. Post-stratification weights were calculated to reflect the age distribution of the 2016 Lesotho census. Additional weights were used for the subsample of women replying to the sexual violence questions.* |
| Study size | 10 | Explain how the study size was arrived at  [Methods, paragraph 1] *The sample size was powered on a relative standard error of 30% for incidence of HIV in adolescent girls and young women (AGYW).* |
| Quantitative variables | 11 | Explain how quantitative variables were handled in the analyses. If applicable, describe which groupings were chosen and why  [Methods, paragraph 4] *Drought was quantified using precipitation estimates from the Climate Hazards Group InfraRed Precipitation with Station data (CHIRPS), which blends a variety of satellite imagery with interpolated weather station data to create gridded rainfall estimates at dekadal time-step and 0.05° resolution, or approximately 30 km2 [20]. To capture the multi-season impact of El Nino, the two-year total rainfall from June 2014 to May 2016 was summed and then ranked among all two-year rainfall amounts within the 1981 to 2016 period and converted to an empirical percentile; therefore, a value of 1% signifies the driest 2-year period in the 35-year record, 100% the wettest, and 50% close to the median value. By using the rainfall accumulation over a 24-month period, the accumulation of data decreased the relative importance of errors in rainfall measurement. Use of these biennial deviations also ensured that any observed negative income shock for that period was a reflection of deviations from the norm, rather than a continuation of underlying farmers’ long-term adjustments to declining precipitation levels [20]. This gridded data set was prepared by the Geospatial Analysis Team at the Analysis and Trends Service (VAM) of the World Food Programme (WFP), using in-house developed scripts.*  *Latitude and longitude data from the centroid of each LePHIA sampled enumeration area (EA) was overlaid on the gridded rainfall dataset, with all individuals within each gridded area assigned the same level of rainfall, using ArcGIS Pro 2.0.1 (ESRI). Drought was defined at a percentile of 15% or lower of the record in order to generate a binary variable that approximates the level below which rainfall deficits are particularly harmful to gross domestic product (GDP) and maize yields [6].*  [Methods, paragraph 6] *A household wealth quintile was generated using principal components analysis of household assets to generate a wealth score, based on the previously described methodology used in the DHS [19]. Poverty was defined as a household living in the lowest two quintiles.* |
| Statistical methods | 12 | (*a*) Describe all statistical methods, including those used to control for confounding  [Methods, paragraph 7] *We used logistic regression to assess the association of drought with individual-level likelihood of infection as a binary variable, stratified by region and gender, based on prior data indicating that income shock differentially impacts males vs females [19, 22], as does urban vs rural location [6]. The analysis was stratified by age, to highlight the 15-24 year olds, and where numbers allowed, into adolescents (aged 15-19) vs. young adults (aged 20-24), to identify those most likely to have been recently infected.*  [Methods, paragraph 8] *A multivariable logistic regression model of the association of drought with HIV prevalence was constructed according to a UNAIDS hierarchical framework linking environmental disasters to population displacement, poverty and behavioral change [3], and included variables known to be associated with HIV infection, namely age and household economic status, or to be independently associated with drought in univariable analysis with a p-value <0.10, and be plausibly associated with HIV infection.*  [Methods, paragraph 9] *Because of the multiple comparisons included in this study, we used the Benjamini-Hochberg method to adjust the probabilities for the chance of a false positive [28]. For this calculation, we obtained the false discovery (FDR) corrected p value for each hypothesis and for each strata of analysis, for instance including all univariable analyses conducted to assess the associations between drought and behavior in rural females. We used an acceptable level of false positives of 5%, and report significance based on the corrected p-value, with a corrected p value of 0.10 considered weak evidence of an association. For the multivariable analysis, we used a significance threshold of 0.025 based on the Bonferroni correction of adjusting the normal p value of 0.05 to reflect the two models.* |
|  |  | (*b*) Describe any methods used to examine subgroups and interactions  [Methods, paragraph 8] *Because there appeared to be opposite patterns of association between drought and HIV prevalence by sex, we fitted the model with an interactive variable combining drought and sex, with participants living without drought coded as 0, males living in drought coded as 1, and females living in drought coded as 2.* |
|  |  | (*c*) Explain how missing data were addressed  The analysis only included complete cases but there was very little missing data. |
|  |  | (*d*) If applicable, describe analytical methods taking account of sampling strategy  [Methods, paragraph 6] *Design weights were calculated based on sampling design, including probability of household selection, and adjusted for non-response at the household, individual and biomarker level using the Chi-Squared Automatic Interactor Detector (SI-CHAID) software (Statistical Innovations); this was stratified by urban or rural residence, age group, and region, with peri-urban populations grouped with rural as per the Lesotho Bureau of Statistics. Post-stratification weights were calculated to reflect the age distribution of the 2016 Lesotho census. Additional weights were used for the subsample of women replying to the sexual violence questions.*  [Methods, paragraph 6] *All analyses were done in Stata version 15.1 using weighted data, with Jackknife replicate weights used for variance estimation.* |
|  |  | (*e*) Describe any sensitivity analyses  There were no sensitivity analyses done except as part of weighting. |
| Results | | |
| Participants | 13 | (a) Report numbers of individuals at each stage of study—eg numbers potentially eligible, examined for eligibility, confirmed eligible, included in the study, completing follow-up, and analysed  [Results, paragraph 1] *Of 9403 selected households, 8824 (94%) completed a household interview; 9% of households in rural areas were vacant, compared to 6% of urban households. Of 7893 eligible women and 6135 eligible men, 12887 (92%) completed an interview, and 11682 of those (91%) were tested for HIV.* |
|  |  | (b) Give reasons for non-participation at each stage  [Results, paragraph 1] *Of 9403 selected households, 8824 (94%) completed a household interview; 9% of households in rural areas were vacant, compared to 6% of urban households. Of 7893 eligible women and 6135 eligible men, 12887 (92%) completed an interview, and 11682 of those (91%) were tested for HIV.* |
|  |  | (c) Consider use of a flow diagram  [Results, paragraph 1] This reports the flow of participants.  *Of 9403 selected households, 8824 (94%) completed a household interview; 9% of households in rural areas were vacant, compared to 6% of urban households. Of 7893 eligible women and 6135 eligible men, 12887 (92%) completed an interview, and 11682 of those (91%) were tested for HIV.* |
| Descriptive data | 14 | (a) Give characteristics of study participants (eg demographic, clinical, social) and information on exposures and potential confounders  [Results, paragraph 1] *The vast majority of households (94.8% of urban and 93.9% of rural) were in areas of drought (Fig 1). There were no differences in the sex ratio in drought versus non-drought areas, nor was there a difference in age distribution (Table 1). Compared to all urban residents and those living in rural non-drought areas, a greater proportion of participants in rural drought-affected areas were in impoverished (55.3%) and food insecure households (39.6%).*  [Results, figure 1] ***Figure 1. Map of Precipitation in Lesotho during the Drought of 2014-2016 relative to 1981-2016***  [Results, table 1] ***Table 1. Selected Characteristics of Adult Participants (15-59 years) by Residence and Drought Status, Lesotho 2016-2017*** |
|  |  | (b) Indicate number of participants with missing data for each variable of interest  [Results, table 1] ***Table 1. Selected Characteristics of Adult Participants (15-59 years) by Residence and Drought Status, Lesotho 2016-2017*** |
| Outcome data | 15 | Report numbers of outcome events or summary measures  [Results, paragraph 4, table 3*] HIV prevalence was 26.9% (95% CI 25.4-33.4%) in urban areas and 24.7% (95% CI 23.5-25.8%) in rural areas, and was higher in females (Table 3).* |
| Main results | 16 | (*a*) Give unadjusted estimates and, if applicable, confounder-adjusted estimates and their precision (eg, 95% confidence interval). Make clear which confounders were adjusted for and why they were included  [Results, table 2] ***Table 2. Associations between Drought and Selected Characteristics by Residence and Sex, and on Youths aged 15-24 Years, Lesotho 2016-2017***  [Results, table 3] ***Table 3. Associations between Drought and HIV Prevalence and Care by Residence, Sex and Age, Lesotho 2016-2017*** |
|  |  | (*b*) Report category boundaries when continuous variables were categorized  [Methods, paragraph 5] *Drought was defined at a percentile of 15% or lower of the record in order to generate a binary variable that approximates the level below which rainfall deficits are particularly harmful to gross domestic product (GDP) and maize yields [6].*  [Methods, paragraph 6] *A household wealth quintile was generated using principal components analysis of household assets to generate a wealth score, based on the previously described methodology used in the DHS [19]. Poverty was defined as a household living in the lowest two quintiles.*  [Methods, paragraph 7] *To assess the impact on the treatment cascade, we assessed the association of drought with awareness of HIV status, reported ART use, and VLS, defined as HIV RNA<1000 copies/ml in PLHIV.* |
|  |  | (*c*) If relevant, consider translating estimates of relative risk into absolute risk for a meaningful time period  Not relevant. |
| Other analyses | 17 | Report other analyses done—eg analyses of subgroups and interactions, and sensitivity analyses  [Results, table 4] ***Table 4. Associations between Drought and Related Characteristics, and HIV Prevalence, in 15-24 and 25-59 year olds, Lesotho 2016-2017*** |
| Discussion | | |
| Key results | 18 | Summarise key results with reference to study objectives  [Discussion, paragraphs 1-2] *The LePHIA survey provided an opportunity to assess coping mechanisms that might have been adopted in the 12 months prior to the survey, during times of crop failure and hunger. The findings from our study suggest a drought response pattern including alteration of behaviors increasing risk [6]. The observed associations were stronger in rural areas, where food shortages and income shocks would be most pronounced due to limited diversification of economic activity.* |
| Limitations | 19 | Discuss limitations of the study, taking into account sources of potential bias or imprecision. Discuss both direction and magnitude of any potential bias  [Discussion, paragraph 5] *Study limitations include the ecological nature of the study, which did not measure indicators of the experience of drought at the individual level. However, we were able to demonstrate that households in rural drought areas were significantly more likely to be impoverished, which we did not observe in urban areas, supporting the hypothesized impact of drought on agrarian households. Second, the timing of the survey, which was conducted after the drought, and its cross-sectional design, limit the ability to determine causality for observed associations; however, the focus on behavior changes in the past year makes it more likely that observed behaviors occurred during the drought. Social desirability bias may have influenced reporting of sexual behaviors, although under-reporting would be more likely to bias the results toward the null hypothesis. The severity of the drought also meant that few people were unaffected, limiting our ability to assess certain outcomes. Furthermore, the drought data were based on combined satellite and ground station measurement of rainfall, and there were only two ground stations in Lesotho with intermittent functionality during the drought period. This means that the drought grid was derived primarily from statistical downscaling of satellite data, impacting the accuracy of estimates at high resolution and allowing for some misclassification of drought severity. However, the use of two years of data and using relative rank rather than an absolute value for our indicator of drought should ensure that our results are reasonably robust. It should also be noted that the survey only included persons residing in Lesotho; those who had left and not returned as of the survey date may have influenced HIV transmission, but we could only observe the association between drought, migration, and HIV infection among returned migrants. Although there was only weak evidence of an association between migration and drought for rural males, the multivariable model identified a strong association between migration and HIV infection in young people. Migration has played a key role in the epidemic in the region, as male migrants have been reported to engage in commercial sex, and have less access to HIV care [25,37,38]. The lower prevalence of HIV in young men suggests that, in the absence of migration, drought and its attendant financial constraints might protect men as it may reduce both incentives and resources to pay for commercial or transactional sex [20]. Finally, due to the breadth of this analysis, the multiple comparisons increase the risk of Type I errors, which we have tried to limit by using FDR. Some of the multivariate findings that were marginally significant before the correction become non-significant with the application of FDR. In part, this is due to the smaller number of cases once the data are disaggregated, limiting the statistical power to test multiple associations at once. Future work with larger samples of young people may enable more fine analyses of causality and the associations between risk factors. However, the positive and statistically significant associations between drought and risk behaviors among young women remain, and between those behaviors and HIV infection, presenting a plausible and concerning pattern of vulnerability..* |
| Interpretation | 20 | Give a cautious overall interpretation of results considering objectives, limitations, multiplicity of analyses, results from similar studies, and other relevant evidence  [Discussion, paragraphs 2-3] *The findings from our study suggest a drought response pattern including alteration of behaviors increasing risk [6]. The observed associations were stronger in rural areas, where food shortages and income shocks would be most pronounced due to limited diversification of economic activity. They are consistent with other results from LePHIA, which found some association between food insecurity and incident infection in adolescent girls and young women [29]. Moreover, we found that there was an increase in the constellation of risk behaviors which were independently associated with HIV infection, including transactional and commercial sex, suggesting that some women may indeed be relying on sexual favors to survive drought, if less openly in rural areas than in urban [19, 30]. The increase in early sexual debut and reduced educational attainment in girls in rural areas is consistent with adolescent girls being removed from school for transactional partnerships or marriage, so that families can benefit from the bride price [31]. The strength of the association between marriage and HIV infection suggests that there may be relatively high rates of transmission between married couples, either through infections developed prior to marriage or through infections acquired from extra-marital partners.*  *For women in urban areas, despite an attenuated effect of the drought on household poverty, being in a drought-affected region was associated with substantially higher reporting of commercial and coercive sex, which supports results from a recent Populations Fund (UNFPA) survey in Lesotho linking drought to an increase in gender-based violence [32]. This is indicative of increased vulnerability, potentially reflective of internal migration from rural areas seeking employment, and of global disruption of the economy due to the severity of drought and impact on other sectors [33, 34]. As 48% of women reporting a history of commercial sex work in Lesotho are HIV-positive and forced sex also increases the risk of HIV [18], the circular migration back to rural homesteads poses significant risk to partners and communities [35, 36]. Furthermore, as education was strongly protective against HIV infection in our multivariable model, the lower school enrolment seen in young rural females in drought areas could have far-reaching consequences, both in terms of HIV acquisition as well as entrenchment of poverty. Drought clearly has both immediate and long-term consequences, and requires different targeted policies.*  *In terms of the HIV continuum of care, the results are reassuring in terms of broader epidemic control, as there were no observed associations in terms of VLS. There were various interventions in the form of food support and household rations, including the Super Cereal from Global Fund and Ready to Use Therapeutic Food from the WFP, targeting malnourished PLHIV on ART or with tuberculosis, and these efforts may have been successful in incentivizing PLHIV to stay on treatment and in mitigating the worst hunger- associated effects of the drought. However, the highly migratory nature of the Lesotho population makes it difficult to interpret the completeness of the data; as those on ART were preferentially provided with food support [13], stable patients might have been more likely to remain in the country, with non-adherent or undiagnosed PLHIV not being captured by the survey due to out-migration.* |
| Generalisability | 21 | Discuss the generalisability (external validity) of the study results  [Discussion, paragraph 6] *In conclusion, this study provides further evidence for the need for a coordinated policy and strategy to attenuate the effects of drought on HIV infection in Southern Africa. Young women appear particularly susceptible to the negative income shocks of drought, whereas men seem predominantly affected in terms of labor migration and its potential for increased long-term risk [39]. Potential interventions should minimize these shocks by targeting the myriad factors contributing to vulnerability, and could include cash transfers to encourage families to keep children in school and avoid early marriage, provided to rural families in times of food shortage [40,41], and expanded programs for adolescent girls and young women, sex workers and migrants, including pre-exposure prophylaxis (PrEP). In light of the anticipated acceleration of such climatic extremes, more research is urgently needed on improving resilience of crops to drought, to mitigate the severity of impact on household incomes and public health.* |
| Other information | | |
| Funding | 22 | Give the source of funding and the role of the funders for the present study and, if applicable, for the original study on which the present article is based  [supporting information] *This project has been supported by the U.S. President’s Emergency Plan for AIDS Relief (PEPFAR) through the Centers for Disease Control and Prevention (CDC) under the terms of cooperative agreement #U2GGH001226. The findings and conclusions are those of the authors and do not necessarily represent the official position of the funding agencies.* |

*Give information separately for exposed and unexposed groups.

**Note:** An Explanation and Elaboration article discusses each checklist item and gives methodological background and published examples of transparent reporting. The STROBE checklist is best used in conjunction with this article (freely available on the Web sites of PLoS Medicine at http://www.plosmedicine.org/, Annals of Internal Medicine at http://www.annals.org/, and Epidemiology at http://www.epidem.com/). Information on the STROBE Initiative is available at www.strobe-statement.org.
